# Genomic diversity and functional adaptation of *Limosilactobacillus reuteri* isolated from diverse ecological niches

**DOI:** 10.3389/fmicb.2025.1732127

**Published:** 2025-12-12

**Authors:** Yuexin Sun, Qian Zhao, Weicheng Li, Lai-Yu Kwok, Heping Zhang

**Affiliations:** 1 Key Laboratory of Dairy Biotechnology and Engineering (lMAU), Ministry of Education, Inner Mongolia Agricultural University, Hohhot, China; 2 Key Laboratory of Dairy Products Processing, Ministry of Agriculture and Rural Affairs, Inner Mongolia Agricultural University, Hohhot, China; 3 Inner Mongolia Key Laboratory of Dairy Biotechnology and Engineering, Inner Mongolia Agricultural University, Hohhot, China; 4 Collaborative Innovative Center for Lactic Acid Bacteria and Fermented Dairy Products, Ministry of Education, Inner Mongolia Agricultural University, Hohhot, China; 5 College of Food Science and Engineering, Inner Mongolia Agriculture University, Hohhot, China

**Keywords:** *Limosilactobacillus reuteri*, comparative genomics, antibiotic resistance genes, bacteriocin, CRISPR-Cas system, lactic acid bacteria

## Abstract

*Limosilactobacillus reuteri* is a widely utilized probiotic, however, the genomic diversity and evolutionary mechanisms underlying its adaptation to various hosts and environments remain incompletely understood. This study employed comparative genomics to analyze 176 *L. reuteri* genomes from animal (rodents, mammals, ruminants, and birds), human intestinal, and food sources (dairy products, fermented foods; 89 newly sequenced and 92 retrieved, 5 excluded by ANI < 95%). We assessed genomic features, average nucleotide identity, pan/core genomes, carbohydrate-active enzymes, bacteriocin production, CRISPR-Cas systems, and antibiotic resistance genes. The pan-genome consisted of 16,814 genes, while the core genome contained 553 genes. Core-gene phylogeny revealed seven clades, rodents isolates were positioned closer to the root. The clustering trend of fermented foods isolates in the phylogenetic tree may indicate that these strains have undergone convergent evolution or adaptive evolution in a specific environment. CAZymes varied across sources, and the predicted bacteriocin clusters were enriched in animal-derived, particularly in rodent isolates. CAZy functional composition in *L. reuteri* is shaped by the ecological niche and host environment, reflecting a pattern of host-driven evolutionary adaptation. CRISPR–Cas systems were present in 23.3% of genomes, predominantly in rodents isolates, indicating strong anti-phage capabilities. The heterogeneity of CRISPR-Cas systems among sources suggests that subpopulations of *L. reuteri* have been subjected to different evolutionary pressures. The predominance of Type II systems agrees with their widespread occurrence in *lactobacilli*. The presence of multiple probiotic function-related genes across all separation sources confirms the robust probiotic potential of *L. reuteri.* Antibiotic resistance genes, including *tet*, *erm*B, and *vat*E, were most prevalent among animal-derived isolates, with the highest numbers occurring in mammals and the lowest in rodents. Therefore, strain-specific safety assessments are necessary prior to clinical or food applications. The findings underscore the significance of host-specific adaptations in shaping the genetic and functional profiles of *L. reuteri*, offering valuable implications for its application in food-derived, human-derived, animal-derived and therapeutics.

## 1 Introduction

Probiotics are living microorganisms that confer health benefits to the host when consumed in adequate quantities (Suez et al., 2019). Current Research on probiotics has advanced the management of diseases, facilitated metabolic modulation, and provided insights into antimicrobial resistance. Nevertheless, challenges such as strain specificity, resistance risks, and clinical translation persist and necessitate rigorous evaluation. Numerous clinical trials have demonstrated that probiotics can modulate gut microbiota and enhance host outcomes in various intestinal conditions (Fasiha Fayyaz Khan et al., 2025; Mazhar et al., 2025).

*Limosilactobacillus* (*L.*) *reuteri* is a globally recognized probiotic lactic acid bacterial species (Buron-Moles et al., 2019). As a natural inhabitant of the human gastrointestinal tract, *L. reuteri* has been studied for benefits such as enhancing immune function, improving nutrient absorption, and maintaining intestinal mucosal integrity (Zhao et al., 2019; Fernández et al., 2020). Its ability to colonize diverse environments and hosts has made it a model organism for studying host–microbe interactions and evolutionary adaptations.

The taxonomic classification of *L. reuteri* has evolved over time. Initially identified as *Lactobacillus reuteri* by Kandler et al. (1980), it was reclassified into the genus *Limosilactobacillus* in 2020 (Zheng et al., 2020). This species is widely distributed across various environments, including the intestines of humans and animals, as well as dairy products, fermented dough, and meat products (Abuqwider et al., 2022). The quantity of *L. reuteri* in the human gut varies depending on factors such as host type, age, or gut location (Duar et al., 2017b). Its ability to colonize a range of vertebrates, including pigs, rodents, and chickens, highlights its adaptability and evolutionary success in diverse host environments (Crepinsek et al., 2020).

*L. reuteri* exhibits remarkable genetic diversity, which is closely linked to its host and habitat (Oh et al., 2010). Li et al. revealed host-specific evolution through phylogenetic genomics. Among them, the colonization ability of rodent strains in the mouse intestine was significantly better than that of other host-derived strains, supporting host-driven adaptive evolution (Li et al., 2023). Frese et al. demonstrated the host-specific evolution of *L. reuteri* using germ-free mice, identifying hundreds of host-specific genes in rodent and human isolates (Frese et al., 2011). Their comparative genomic analysis of 57 isolates from six hosts revealed stronger genome adaptability in rodent isolates, while human isolates exhibited a more streamlined evolutionary process. Similarly, Wegmann et al. highlighted the host-specific nature of *L. reuteri* genomes, particularly in genes encoding cell surface proteins, which may explain the formation of two distinct phylogenetic branches (Wegmann et al., 2015). Yu et al. further contributed to this understanding by sequencing 16 *L. reuteri* isolates from Inner Mongolia, underscoring the link between genomic diversity and ecological adaptation (Yu et al., 2018). Although essential breakthroughs have been made in the research of the genetic characteristics of *L. reuteri* in recent years, the evolutionary mechanisms that are driving the genomic diversity and functional adaptation of *L. reuteri* across different habitats, hosts, and environments remain incompletely understood.

To address these gaps, this study employs comparative genomics to explore the phylogenetic relationships, functional gene content, and ecological adaptations of *L. reuteri* isolated from diverse sources. We use comparative genomics to examine phylogeny, functional gene content, and ecological adaptations of *L. reuteri* from diverse sources, including nutrient metabolism genes, carbohydrate-active enzymes, and putative host-associated traits. Our findings revealed distinct functional gene profiles among isolates, reflecting their adaptation to specific habitats. Furthermore, isolates from the same source exhibit apparent phylogenetic clustering, indicating a shared evolutionary history. The study of *L. reuteri* in different habitats not only helps to reveal its adaptability and functional diversity but also enhance our understanding of the evolutionary mechanism of *L. reuteri.* These insights provide a foundation for future studies on probiotic applications and the ecological significance of *L. reuteri*.

## 2 Methods

### 2.1 *Limosilactobacillus reuteri* isolates and genomes

This study sequenced the genomes of 89 *L. reuteri* isolates obtained from animal, food, and human intestinal sources, provided by the Lactic Acid Bacteria Collection Center at Inner Mongolia Agricultural University. *L. reuteri* isolates were activated, cultured, and genomes sequenced. The specific strain information is shown in Supplementary Table S1. The data that support the findings of this study have been deposited into National Genomics Data Center (Ma et al., 2025), Beijing Institute of Genomics (Members C.-N. and Partners, 2025), Chinese Academy of Sciences/China National Center for Bioinformation, under accession number PRJCA042243 that is publicly accessible at https://ngdc.cncb.ac.cn/gwh. Additionally, genome data for 92 *L. reuteri* were retrieved from the National Center for Biotechnology Information (NCBI) database (as of October 2023). Combining these datasets resulted in a total of 181 genomes (Supplementary Table S2), comprising 55 of animal origin, 85 of food origin, 36 of human intestinal origin, and 5 of unknown origin. More specifically, 65 isolates were from dairy products, 35 from human intestinal tract, 21 from rodents, 17 from fermented foods, 15 from mammals, 11 from ruminants, 8 from birds, and 9 from other sources (including 3 from fruits, 1 from fish, and 4 from unknown sources).

A total of 181 publicly available or newly sequenced *L. reuteri* genomes were initially retrieved. We used CheckM (version 1.1.2) to assess the integrity and contamination of each genome, with a completeness level greater than 95% and a contamination level below 5%, with all parameters set to default values. Average nucleotide identity was calculated against the *L. reuteri* type strain, and genomes with ANI < 95% were excluded, consistent with commonly applied prokaryotic species delineation thresholds. After applying all quality and taxonomic filters, 176 high-quality genomes remained and were used for all subsequent comparative analyses.

### 2.2 Activation and culture of bacteria

The isolates, stored in ampoules, were initially inoculated into 5 mL of de Man Rogosa and Sharpe broth (Oxoid, United Kingdom) and cultured at 37 °C for 24 h. After two generations of subculturing, the bacterial cultures were expanded in 50 mL of the same culture medium for genomic DNA extraction.

### 2.3 DNA extraction

Bacterial cells were washed twice with phosphate-buffered saline, and genomic DNA was extracted using a bacterial genomic DNA extraction kit (TIANGEN, Beijing, China) following the manufacturer’s instructions.

### 2.4 Whole-genome sequencing and assembly

A sequencing library was constructed for each isolate, and paired-end sequencing (PE150) was performed using the Illumina NextSeq 2000 platform (Novogene, Beijng, China). Raw reads underwent quality control to remove low-quality data and adapter sequences. High-quality clean-data were assembled into contigs using SOAPdenovo (v2.04; Xie et al., 2014), followed by single-base correction. Gaps in the assembled genomes were filled using GapCloser.^1^

### 2.5 Calculation of average nucleotide identity

Average nucleotide identity was calculated using fastANI software (Jain et al., 2018) to assess genetic diversity and species delineation. A fragment length of 1,000 bp was used for comparisons, and ANI values below 95% were considered indicative of different species. The 95% ANI threshold is widely used for bacterial species definition, consistent with the 70% threshold for DNA–DNA hybridization (DDH), and has been widely adopted in taxonomic studies as a conserved threshold for species definition. Applying this threshold ensures that only genuine *L. reuteri* genes are retained (Olm et al., 2020). The resulting ANI matrices were clustered using the unweighted pair group method with arithmetic mean.

### 2.6 Gene prediction and functional annotation

Gene prediction was performed using Prokka software (V1.14.6; Seemann, 2014) and potential probiotic-related genes were identified. Functional annotation of the core and pan-genome sets was conducted using the Clusters of Orthologous Groups (COG) database (Huerta-Cepas et al., 2017).^2^ Differences in COG functional profiles among genetic lineages were analyzed to explore functional genomic variation.

### 2.7 Pan-genome analysis

The pan-genome of *L. reuteri* was analyzed using Roary software (v3.6.1; Page et al., 2015), which constructed core and accessory gene sets at the subspecies level. A minimum BLASTP protein identity of 95% (−i 95) was used to cluster genes into orthologous groups. The core genome was stringently defined as genes present in 100% of the analyzed genomes (−cd 100). Roary was run using the following parameters: -e --mafft -p 4 -r -t 11 -cd 100, the remaining parameters are the default values.

### 2.8 Phylogenetic tree construction

Phylogenetic trees were constructed using the Neighbor-Joining method based on the core gene set sequences with or without using three strains of *Limosilactobacillus fermentum* as the outgroup (strain ID is EFEL6800, ATCC 14931, DSM20052). The tree was visualized using the Interactive Tree of Life (iTOL) platform,^3^ enabling comparison of genetic backgrounds and evolutionary relationships among the *L. reuteri* genomes.

### 2.9 Carbohydrate-active enzyme analysis

The HMMER package (Minoru et al., 2016) was used to annotate CAZyme in the genome sequences, with reference to the CAZyme database.^4^ Detailed CAZyme family information was retrieved from the CAZy database.^5^

### 2.10 Drug resistance genes

Drug resistance genes were identified using the ResFinder database^6^ with thresholds of > 90% sequence similarity and > 60% coverage. Virulence factors were annotated using the VirulenceFinder database^7^ with the same thresholds (Marchin et al., 2005). Additionally, virulence factors of 176 *L. reuteri* genomes were detected by the Virulence Factor Database.^8^

### 2.11 Bacteriocin prediction

Secondary metabolite synthesis gene clusters, including those encoding bacteriocins, were predicted using the Prediction Informatics for Secondary Metabolomes (PRISM) online tool (Krug and Muller, 2014) and the BAGEL4 online platform (van Heel et al., 2018).

### 2.12 CRISPER-Cas system analysis

The CRISPR-Cas online (Grissa et al., 2007) was used to search for CRISPR arrays and Cas genes in 176 *L. reuteri* genomes. The number of CRISPR gene clusters, Cas gene types, and spacer sequences was recorded. Variations in CRISPR-Cas systems among these genomes were analyzed to explore their diversity and functional implications.

### 2.13 Statistical methods

We performed the Shapiro–Wilk normality test and the Levene homogeneity of variance test. ANOVA was used for comparisons between groups, with a post-hoc Tukey HSD test; non-parametric tests (Kruskal-Wallis test, with a post-hoc Dunn test) were used where necessary. Benjamini-Hochberg correction was used for multiple comparisons. Effect sizes (Hedges’ g) and their 95% confidence intervals are given. All analyses were performed in R (v4.5.0).

## 3 Results

### 3.1 Characteristics of *Limosilactobacillus reuteri* genomes from different sources

**Figure 1:**
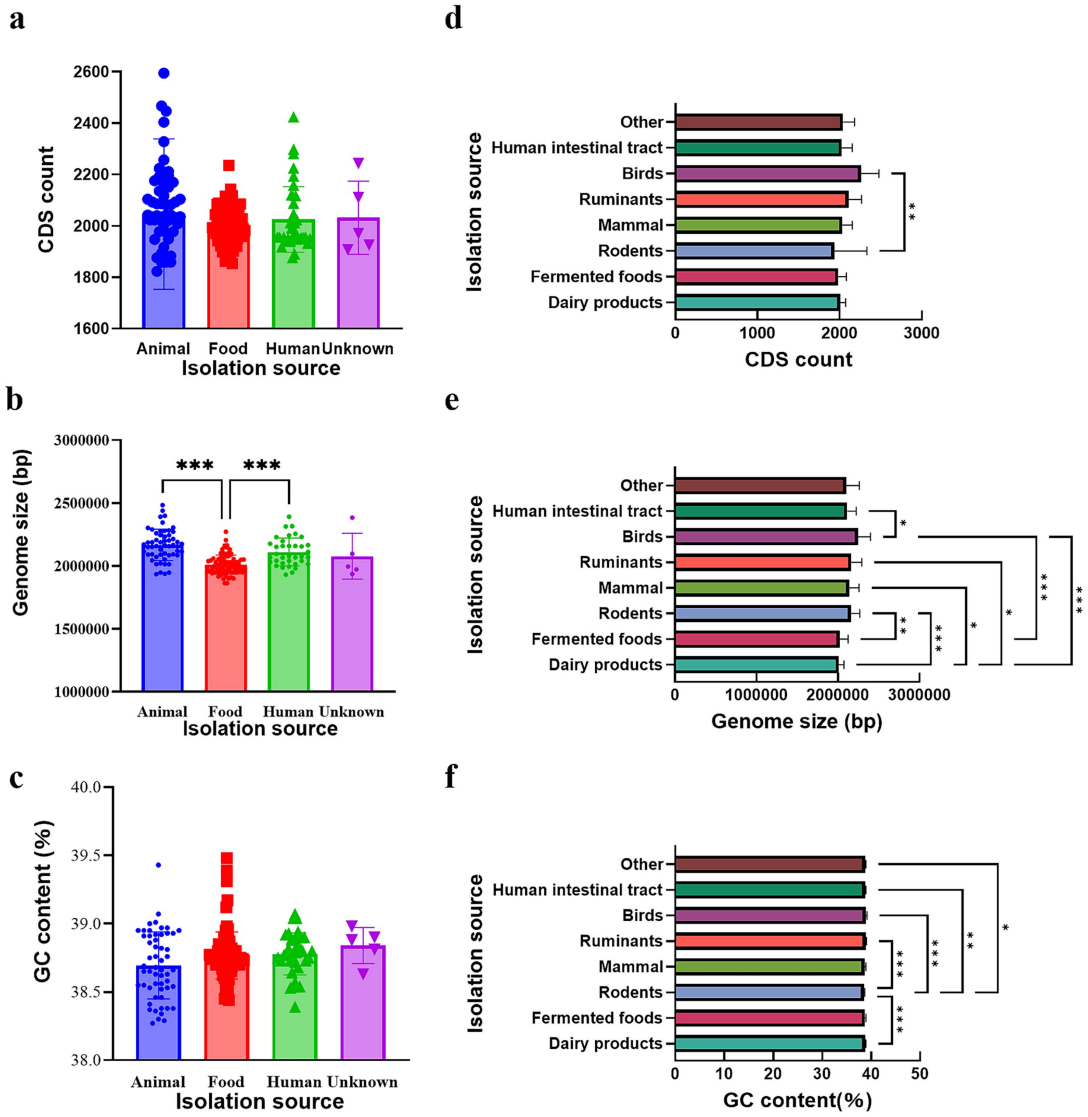
Comparison of basic genomic characteristics of *Limosilactobacillus reuteri* isolates from different sources. **(a)** Number of coding sequences (CDS), **(b)** genome size, and **(c)** GC content. The bar charts illustrate genomic features among isolates from animal, human intestinal, and food sources, reflecting their adaptive evolution to distinct ecological niches. **(d)** Number of coding sequences (CDS), **(e)** genome size, and **(f)** GC content in dairy products, fermented foods, rodents, mammals, ruminants, birds, the human intestinal tract, and other environments. Data are presented as mean ± standard error (SE). Statistical significance was evaluated using one-way ANOVA followed by Tukey’s *post-hoc* test. Asterisks indicate significant pairwise differences: *p < 0.05, **p < 0.01, ***p < 0.001.

The *L. reuteri* genomes sequenced and assembled in this study exhibited high quality, these results confirm the reliability of the sequencing and assembly data for downstream bioinformatics analysis. The 176 *L. reuteri* genomes analyzed in this study exhibited an average genome size of 2.08 ± 1.23 Mb, a GC content of 38.75% ± 0.20%, and an average scaffold number of 143.6 ± 129.3. The reference strain *L. reuteri* DSM 20016^T^, isolated from the intestine of an adult human, served as the type strain for comparison (Sriramulu et al., 2008). Its genome size and GC content were consistent with data available in the NCBI database, validating the accuracy of our sequencing and assembly pipeline. Comparative analysis of genomic characteristics across isolates from different regions and sources revealed significant differences in coding sequence counts and genome size (*p* < 0.05; Figure 1). Foodborne strains exhibited smaller genomes and higher GC content, likely due to gene loss associated with host adaptation (Sheppard et al., 2018). In contrast, animal-derived isolates had significantly larger genomes (*p* < 0.001), while human intestinal-derived isolates showed intermediate genome sizes but fewer coding sequences. Genome size is a fundamental biological characteristic that exhibits variation among species and even within strains of the same species (Westoby et al., 2021). This variation is influenced by environmental factors and evolutionary processes (Ngugi et al., 2023). Generally speaking, the more complex the environment in which the strain is located, the larger the genome size, and the less the gene decay experienced (Kraaijeveld, 2010). No significant differences in GC content were observed among isolates from different sources.

We then compared the genomic characteristics of isolates obtained from eight ecological sources, including dairy products, fermented foods, rodents, mammals, ruminants, birds, the human intestinal tract, and miscellaneous environments were compared (Figures 1d–f). A substantial degree of variation was observed in genome size, GC content, and protein-coding sequence (CDS) numbers among the various ecological groups. The genome size ranged from approximately 1.8 to 2.2 Mbp. Strains isolated from dairy products and fermented foods exhibited significantly smaller genomes compared to those from rodents, mammals, ruminants, and birds (*p* < 0.05 to *p* < 0.001). Strains from host-associated environments, ecially rodents and mammals tended to have larger genomes, whereas human intestinal tract isolates displayed intermediate genome sizes. Similarly, the GC content showed significant ecological stratification, ranging from approximately 38.5 to 40.5%. Isolates from dairy products and fermented foods had the lowest GC content, while rodent and mammalian isolates showed the highest GC content (*p <* 0.001). Intermediate GC content values were observed in isolates from birds and ruminants. The number of predicted CDSs mirrored genome size trends. Dairy-derived isolates possessed the lowest CDS counts, whereas host-adapted groups rodent and mammal isolates had significantly higher CDS counts (*p <* 0.01). Only minor variation in CDS numbers was observed among isolates from birds and the human intestinal tract, which formed a middle range between host-adapted and food-origin groups.

These findings suggest that *L. reuteri* has undergone distinct evolutionary processes to thrive in its respective environments. The larger genome size of animal-derived isolates (*p* < 0.05) may reflect their need for additional genetic flexibility to adapt to diverse host conditions. Overall, the observed genomic variations among *L. reuteri* strains are likely influenced by a combination of environmental factors, natural selection, genetic variation, and host-specific lifestyles. Because these source-related genome size and CDS differences were apparent at the whole-genome level, we next examined whether the same ecological partitioning could also be detected at the nucleotide identity level using ANI.

### 3.2 Average nucleotide identity analysis

**Figure 2:**
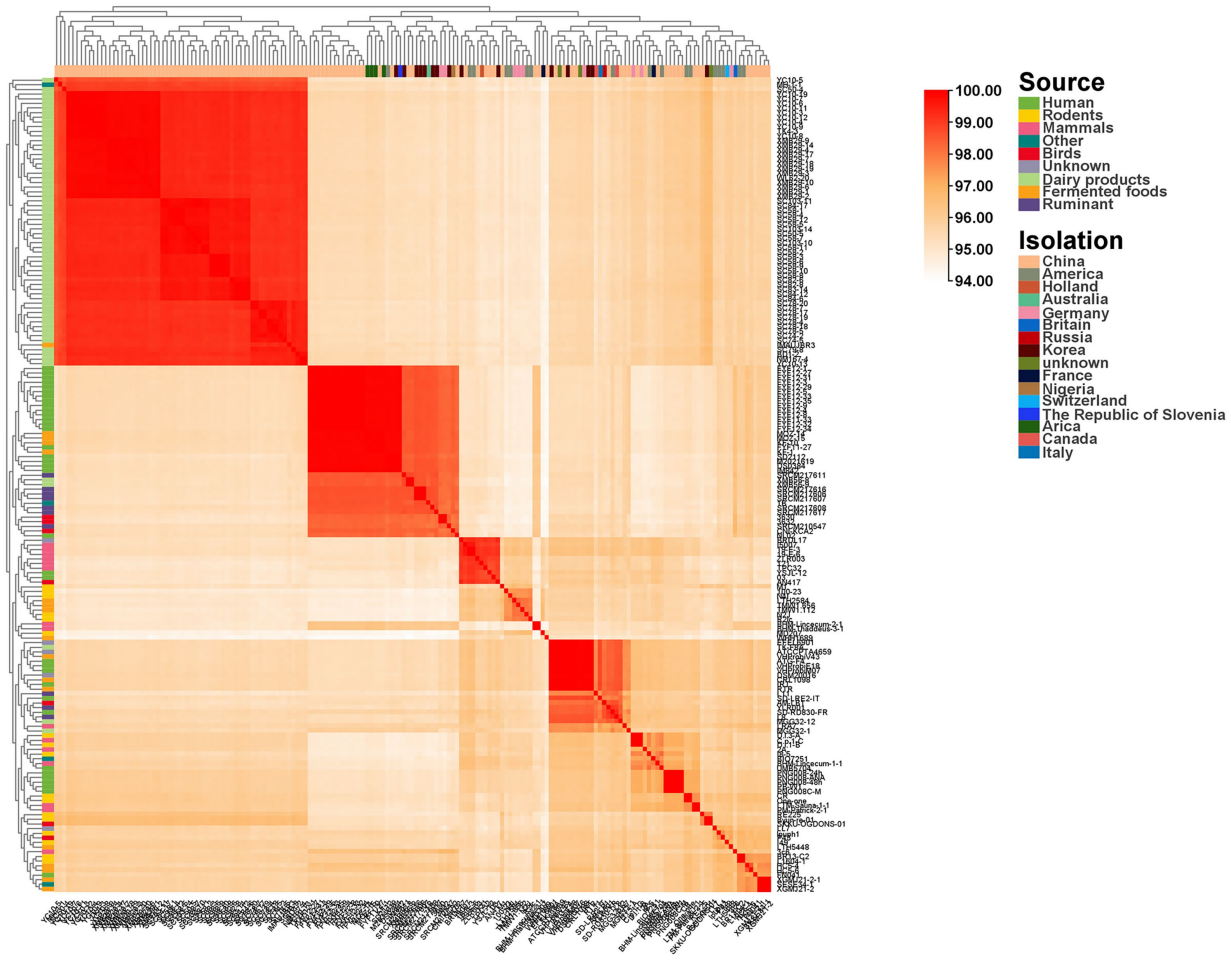
Average nucleotide identity (ANI). Average nucleotide identity (ANI) heatmap and hierarchical clustering of 176 *L. reuteri* genomes across ecological sources. Pairwise ANI values are shown as a heatmap with agglomerative clustering on rows and columns. Left bars annotate Source (Rodents, Mammals, Ruminants, Birds, Dairy products, Fermented foods, Human intestinal tract, Other).

The results of the ANI analysis conducted on the genomes of *L. reuteri* isolates, including a comparison with the type strain DSM20016^T^, are shown in Figure 2. The ANI values across all genomes averaged 96.33% ± 1.59%. Following the species delineation threshold proposed by Richter and Rossello-Mora (2009), an ANI range greater than 95% indicates the same species. Based on this criterion, 176 genomes analyzed in this study were confirmed to belong to *L. reuteri*. The five genomes with ANI values below 95% were excluded from further analysis.

The ANI results also revealed a clear clustering pattern among genomes from the same source or geographic location. Genomes from the same isolation source or region exhibited high nucleotide consistency, suggesting a close genetic relationship. This clustering trend highlights the influence of isolation source and geographic origin on the intraspecific genetic diversity. Annotation bars show that isolation source explains most clustering, whereas geographic labels are broadly interleaved, indicating weak phylogeographic structure relative to ecology (Pasolli et al., 2019).

The ANI heat map and hierarchical clustering of 176 *L. reuteri* genomes from different ecological sources showed (Figure 2) that rodents constitute a large cohesive block with uniformly high within-group ANI; narrow dark bands connecting to mammal and human blocks indicate close genomic affinity and host-proximal diversification (Velez et al., 2023). Mammals are distributed in two to three sub-blocks adjacent to rodents with gradual transitions toward rodents and human blocks, compatible with shared ancestry and partial mixing (Li et al., 2023). Ruminants are embedded within a subset block of the mammal and rodents region, exhibiting close similarity and moderate connectivity, suggesting their status as a diet-related sub-structure nested within the mammalian backbone (Yu et al., 2018). Birds constitute a distinct, smaller block with clearer boundaries from the mammal and food block, pointing to a more divergent, host-restricted cohort and limited recent exchange (Zhang et al., 2020). Dairy products form a compact, high-ANI community nested within the mammalian block, suggesting recent differentiation following domestication in stable, nutrient-rich environments (Somerville et al., 2022a). Fermented foods comprise one or two compact communities adjacent to the dairy block; these exhibit high ANI values but slightly increased heterogeneity, consistent with multiple introductions or broader fermentation ecologies (Park and Mannaa, 2025). Human intestinal tract is widely distributed across multiple microbial blocks intersecting with rodent/mammalian blocks, indicating the presence of multiple lineages adapted to humans rather than a single human-specific population (Li et al., 2023). Isolated strains scattered at the periphery of other mixed microbial blocks reflect heterogeneous ecological origins, lacking a single cohesive lineage.

### 3.3 Pan-genome, core-genome

A core gene is a gene that is ubiquitous in all or most strains and is typically linked to fundamental cellular processes such as metabolism, replication, and survival. These genes are highly conserved during species evolution and form the basis of pan-genome analysis.

The core genome of a species comprises conserved homologous genes shared by all strains, which are essential for the biological functions and phenotypic stability of the species (Caputo et al., 2019). Using the Roary result, we analyzed the pan-genome and core-genome of 176 *L. reuteri* isolates (Supplementary Figure S1). The pan-genome consisted of 16,814 genes, while the core-genome contained 553 genes. Supplementary Figure S1 shows that the number of core genes in *L. reuteri* tends to remain stable with an increase in the number of strains, while the number of pan-genes increases with the rise in the number of strains, indicating that the genetic material of the species is open (Rajput et al., 2023).

**Figure 3:**
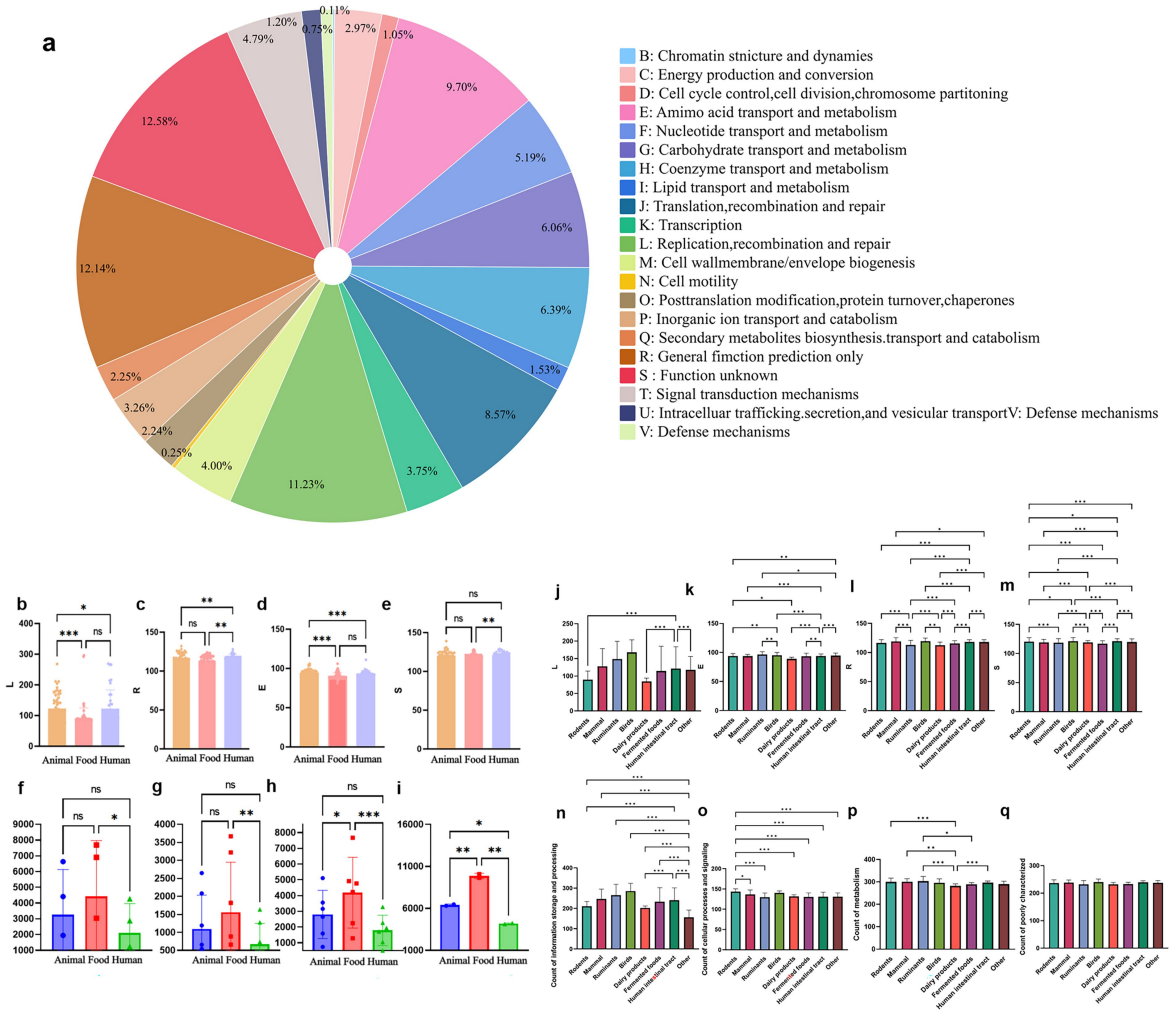
Differences of Clusters of Orthologous Groups (COG) functional annotation of 176 *Limosilactobacillus reuteri* genomes from different sources. **(a)** Pie chart showing distribution of COG annotation results. **(b–i)** COG annotation distribution under eight functional categories: **(b)** replication, recombination, and repair (L); **(c)** general function prediction only (R); **(d)** amino acid transport and metabolism (E); **(e)** function unknown [S]; **(f)** information storage and processing; **(g)** cellular processes and signaling; **(h)** metabolism; and **(i)** poorly characterized. **(j–m)**. COG functional category variation among *L. reuteri* isolates from diverse ecological niches. **(n–q)** Comparison of gene counts assigned to four functional categories: L (Replication, recombination and repair), E (Amino acid transport and metabolism), R (General function prediction only), and S (Function unknown). Error bars represent the standard deviation. Significant differences are indicated (*p < 0.05, **p < 0.01, and ***p < 0.001); ns denotes non-significant. **(j–m)** COG functional category variation among *L. reuteri* isolates from diverse ecological niches.

To elucidate the functional roles of the 553 core genes, we performed functional annotation using the COG database. The COG database, maintained by NCBI, classifies gene products based on homology and facilitates the identification of orthologous genes through extensive protein sequence comparisons. Functional annotation revealed 21 COG categories (Figure 3a). Among these, the top four abundant categories were: function unknown [S] (12.58%), general function prediction only [R] (12.14%), replication, recombination and repair [L] (11.23%), and amino acid transport and metabolism [E] (9.7%). Notably, the distribution of these COG categories varied significantly among isolates from different sources (Figures 3b–e). Generally, genomes from animal and human intestinal sources exhibited a richer diversity of functional genes compared to those from food sources, likely due to differences in ecological niches. COG functional categories that showed significant differences among genomes from different isolate sources included information storage and processing, cellular processes and signaling, metabolism, and poorly characterized across isolation sources (*p* < 0.05; Figures 3f–i). To account for testing several major categories at once, we also ran a multivariate analysis (ANOVA) across these four dominant COG categories, which confirmed a significant overall effect of isolation source (*p* < 0.001).

To explore functional genomic adaptations of *L. reuteri* to different ecological environments, genes were assigned to the COG database and compared across eight isolation sources: rodents, mammals, ruminants, birds, dairy products, fermented foods, the human intestinal tract, and other environments (Figures 3j–m). The different COG functional patterns between isolation sources may further reflect their ecological niche (Xiao et al., 2021). Strains isolated from rodents and birds exhibited the highest number of genes in the L category, significantly exceeding those isolated from dairy products and fermented foods (*p* < 0.001). In contrast, dairy-associated isolates showed the fewest L category genes, suggesting reduced genomic capacity for DNA repair and recombination. In the E category, strains from rodents and mammals contained significantly more genes involved in amino acid metabolism (*p* < 0.01), whereas dairy and fermented food isolates showed lower gene richness in this category. For R and S categories, which reflect accessory and hypothetical gene diversity, host-associated strains (rodents, mammals, ruminants) had significantly higher gene counts compared to dairy and fermented food isolates (*p* < 0.001). These patterns suggest reduced functional diversity in food-origin strains compared to host-associated strains. Figures 3n–q indicates that the annotated genes are distributed across multiple functional COG categories, showing clear variation in abundance. Several categories exhibit prominently higher representation than others. Specifically, categories corresponding to “General function prediction only” and “Function unknown” appear to have the largest bar heights, suggesting that a substantial proportion of annotated sequences are either poorly characterized or involved in broad, multifunctional processes. Moderately represented categories include those related to “Amino acid transport and metabolism,” “Carbohydrate transport and metabolism,” and “Energy production and conversion.” These bars are noticeably taller than those representing specialized functions, indicating their key roles in the metabolic landscape of the organism or sample studied. Conversely, categories linked to cellular structure, defense mechanisms, and signal transduction display relatively lower bar heights, suggesting either lower gene representation or less activity within these functional pathways under the examined conditions.

This highlights the evolutionary plasticity of *L. reuteri* and underscores the importance of ecological context in shaping bacterial functional genomes. These results further underscore the adaptive changes in gene functions that enable *L. reuteri* to thrive in diverse environments. To determine how these source-enriched gene pools correspond to evolutionary lineages, we constructed a core-gene phylogeny of the same 176 genomes.

### 3.4 Phylogenetic analysis based on core-genes

Phylogenetic trees were constructed using 553 core genes from the 176 genomes analyzed with and without using *Limosilactobacillus fermentum* as the outgroup (Supplementary Figure S2). The tree revealed that animal-derived isolates are positioned closer to the root, suggesting they may represent ancestral lineages of *L. reuteri*. Additionally, genomes from similar isolation sources tended to cluster together, indicating a closer genetic relationship. This clustering suggests that the habitats of these isolates have influenced the formation of distinct phylogenetic branches.

Based on the evolutionary relationships depicted in the phylogenetic tree, the 176 *L. reuteri* isolates were divided into two major branches and seven distinct clades (Clade I to VII; Supplementary Figure S2). Clade I consists of 10 isolates, with six derived from food sources and four from animals. Clade II also contains 10 isolates, including seven animal-derived, two human intestinal -derived, and one from an unknown origin. Clade III comprises 19 isolates, which include six food-derived, four animal-derived, six human intestinal-derived, and three from unknown sources. Clade IV consists of 13 isolates, with six food-derived, five animal-derived, one human intestinal-derived, and one from an unknown source. Clade V contains 40 isolates: 14 animal-derived, six food-derived, and 20 human-derived. Clade VI includes 20 isolates, made up of 13 animal-derived, six human-derived, and one food-derived. Finally, Clade VII is the largest, featuring 62 isolates, all of which are food-derived, exhibiting a strong clustering trend. The circular phylogeny resolves several well-defined clades with partial enrichment by isolation source.

Rodent isolates are abundant and form one major clade plus several satellites. Some subclades intermix with human intestinal strains, consistent with host-proximal diversification and occasional host shifts (Duar et al., 2017a; Li et al., 2023). Mammal isolates occur in two to three subclades adjacent to rodent lineages; several cluster tightly with rodent genomes, compatible with shared ancestry or recent cross-host movement (Zhang et al., 2024). Ruminants form small, cohesive sublineages embedded within the broader mammal/rodent backbone, indicating close evolutionary affinity with mammalian gut lineages (Li et al., 2023). Bird-derived genomes form a small, discrete clade with relatively long internal branches, suggesting greater divergence and/or undersampling and limited recent exchange with mammalian or food lineages (Greppi et al., 2020). Dairy products strains concentrate in a compact, shallow subclade nested within mammalian lineages, consistent with recent diversification following domestication (Somerville et al., 2024). Fermented foods are two main clusters are observed: one adjacent to the dairy clade and another embedded among mammalian lineages. Diversity is slightly higher than dairy but still restricted relative to host-associated groups (Somerville et al., 2022b). Human intestinal tract isolates are widely distributed across the tree, including a source-enriched clade and several mixed subclades with rodent and mammal genomes, indicating multiple human-adapted lineages rather than a single human-specific cluster. Other isolates are sparsely scattered at the edges of mixed clades, reflecting heterogeneous origins without a single ecological lineage.

**Figure 4:**
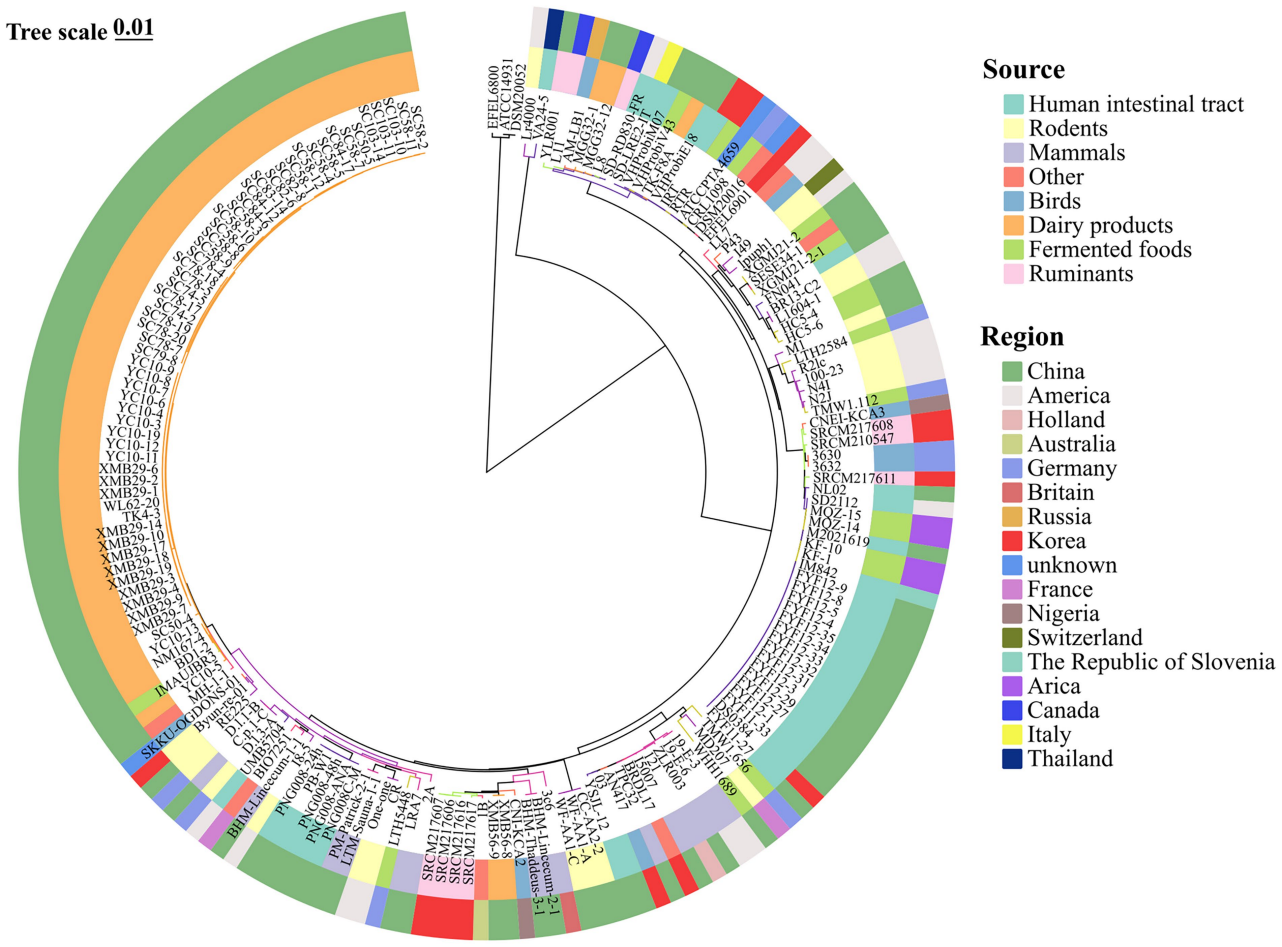
Core-gene phylogenetic analysis of *Limosilactobacillus reuteri* genomes. Phylogenetic tree reconstructed using the Neighbor-Joining method based on 553 core genes. The inner colored ring annotates the isolation source for each strain: Rodents, Mammals, Ruminants, Birds, Dairy products, Fermented foods, Human intestinal tract, and Other. The outer colored ring (“Region”) shows the reported geographic origin for each genome. The first three tips on the tree correspond to three *Limosilactobacillus fermentum* strains (EFEL6800, ATCC 14931, DSM20052) as the outgroup.

The phylogenetic tree also included three *L. fermentum* strains as an outgroup, which showed no clustering with *L. reuteri* genomes, confirming their distant genetic relationship (Figure 4). The type strain DSM20016^T^ clustered with food-derived isolates, while most food-derived strains formed a distinct branch, with a few distributed across other clades. Isolates from similar geographic locations, particularly those derived from animal- and human intestinal-derived sources, exhibited a noticeable aggregation trend, suggesting a link between phylogeny and geographic origin. Notably, sourdough-derived isolates, despite their diverse phylogenetic origins, clustered together, a pattern also observed among food-derived strains in this study. This finding aligns with a previous study reporting significant differences in genome sizes among *L. reuteri* lineages (Zheng J. et al., 2015b). The results demonstrate that genomes from the same isolation source tend to cluster together, likely due to the influence of geographic location and niche-specific adaptations. This source-enriched phylogenetic pattern mirrors the ANI blocks described, confirming that ecological structuring is detectable at both sequence-similarity and tree-based evolutionary scales.

The clustering trend of food isolates in the phylogenetic tree may indicate that these strains have undergone convergent evolution or adaptive evolution in a specific environment, emphasizing the complex interaction between phylogeny, source, and environmental adaptation.

### 3.5 Analysis of CAZymes

Carbohydrate utilization is an important factor in determining the colonization and abundance of *L. reuteri* in the host. Previous sections showed clear ecological structuring of genomes and lineages, we next asked whether CAZymes repertoires follow the same source-related pattern. A total of 72 CAZyme families were predicted across the 176 *L. reuteri* genomes, including 13 carbohydrate-binding modules (CBMs), eight carbohydrate esterases (CEs), 31 glycoside hydrolases (GHs), and 17 glycosyl transferases (GTs). Among these, GHs and CBMs were the most abundant, followed by GTs. The GH family plays a key role in carbohydrate utilization, enabling *L. reuteri* to degrade complex carbohydrates. All 176 *L. reuteri* isolates encode GT2 and GT4, which are mainly synthase enzymes involved in the synthesis of disaccharides such as sucrose, lipopolysaccharides, and cellulose. This suggests that despite differences in isolation sources, the isolates share conserved CAZyme genes, possibly due to common ancestry or horizontal gene transfer.

Notably, the GH73, GH25, and GH13 families were particularly abundant. These families are involved in the degradation of indigestible carbohydrates found in staple foods and dairy products, and GH13 enzymes are involved in starch hydrolysis to hydrolyze digestible polysaccharides (Wei et al., 2023). The CBM family enhances the affinity of GHs for their substrates through a tandem arrangement with GH catalytic domains, facilitating targeted binding to polysaccharides and increasing enzymatic efficiency. The high prevalence of CBM50, which recognizes explicitly lactose, galactose, and their derivatives (Ficko-Blean and Boraston, 2012), underscores the capability of *L. reuteri* to effectively utilize host-derived carbohydrates. This includes breast milk oligosaccharides and mucin, which are typically challenging for the human body to digest.

**Figure 5:**
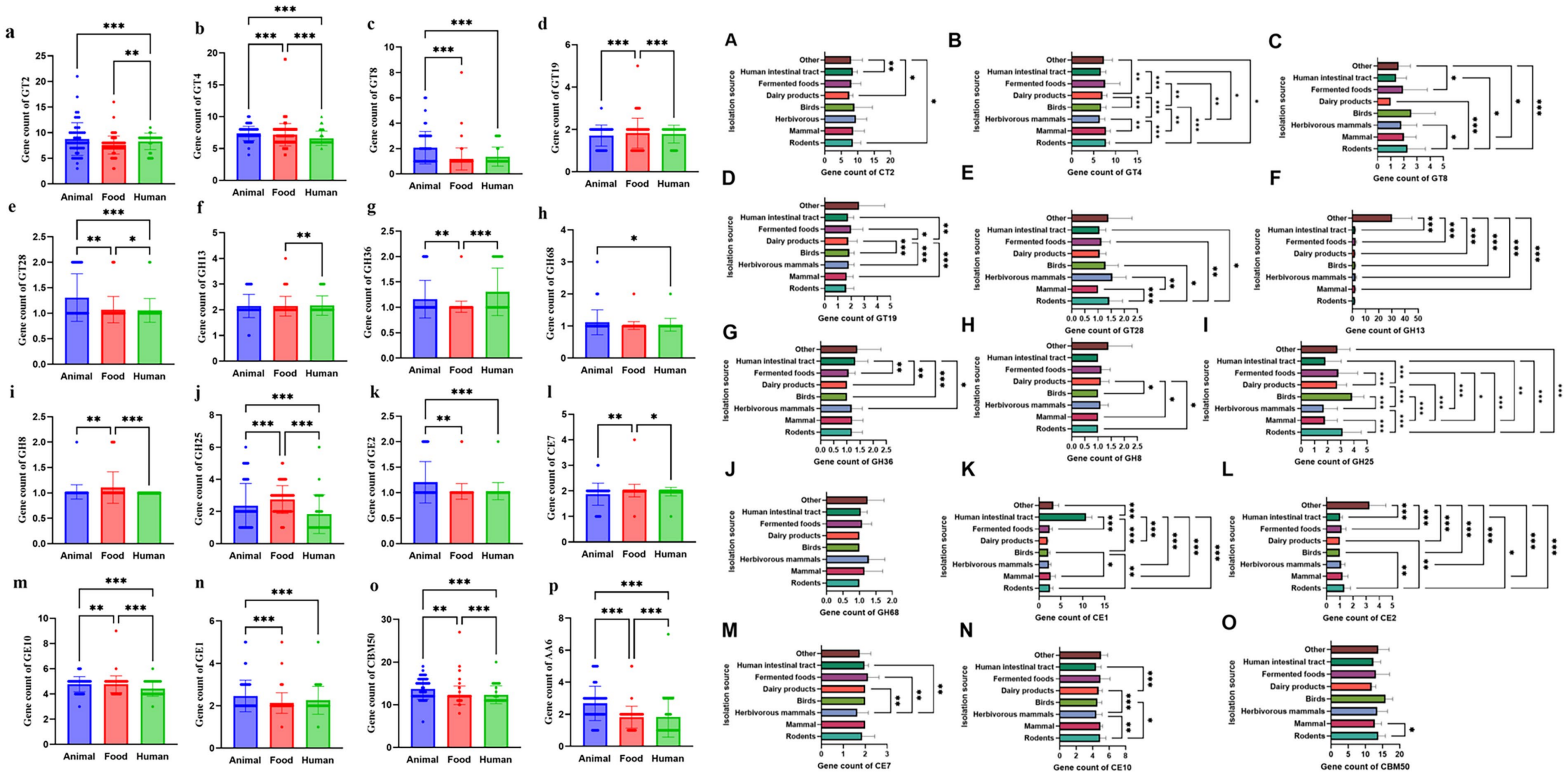
**(a–p)** Number of carbohydrate-active enzyme genes in *Limosilactobacillus reuteri* genomes from different sources. The figure shows the distribution of different CAZyme gene families, including glycosyl transferases (GTs), glycoside hydrolases (GHs), carbohydrate esterases (CEs), and auxiliary activities (AAs). **(A–O)** Distribution of CAZyme classes across *L. reuteri* isolates from distinct ecological sources. Relative abundance of six CAZyme classes—glycoside hydrolases (GHs), glycosyltransferases (GTs), carbohydrate esterases (CEs), polysaccharide lyases (PLs), auxiliary activities (AAs), and carbohydrate-binding modules (CBMs)—in eight ecological groups. Rodent isolates show the highest GH and CE diversity. Dairy and fermented food isolates exhibit marked CAZyme reduction. Error bars represent the standard deviation. Significant differences are indicated (*p < 0.05, **p < 0.01, and ***p < 0.001).

Further analysis revealed significant differences in CAZyme profiles among genomes from different isolation sources (*p* < 0.05; Figure 5). One-way ANOVA indicated that GH13, GH43, GH3, GH36, GT2, GT4, CE10, CE1, and CE2 were all significantly affected by isolation source (*p* < 0.05). Tukey’s HSD multiple-comparison test, as declared in the figure caption, showed that rodent, mammal, and human-intestinal strains formed the high-abundance group, whereas dairy and fermented-food isolates formed the low-abundance group (Tukey-adjusted *p* = 0.000–0.047). Because multiple CAZy families were examined simultaneously, we report the adjusted *p*-value range rather than a single value. Several CAZyme families remained uniquely or predominantly enriched in animal-derived isolates, further supporting host-driven metabolic adaptation. Key differences included variations in GT2, GT4, GT8, GT19, GT28, GH25, GH13, GH68, GH36, GH8, CBM50, CE10, CE1, CE2, and CE7, among others. Additionally, several CAZyme families were uniquely present in specific isolation sources. For example, GT39, GT44, GT11, GT45, GT7, GH30, GH5, GH28, GH115, GH39, and GH67 were exclusively present among animal-derived isolates. GT27 was exclusively identified in food-derived isolates. This enzyme plays a crucial role in the initial step of O-glycan biosynthesis by catalyzing the transfer of N-acetyl-D-galactosamine to serine or threonine residues on protein receptors. Moreover, GT27 demonstrates activity toward the EA2 peptide substrate, although its activity is relatively weak against Muc2 or Muc1b substrates (Wojczyk et al., 2003). AA4, a member of the vanillyl-alcohol oxidase family, is exclusively found in human-derived isolates. It catalyzes the conversion of phenolic compounds that possess side chains at the para-position of the aromatic ring (Gygli et al., 2018).

Comparative CAZy functional classification of *L. reuteri* genomes from eight different isolation sources (Rodents, Mammals, Ruminants, Birds, Dairy products, Fermented foods, Human intestinal tract, and Others) revealed both a conserved core of carbohydrate-active enzymes and marked source-specific functional differences (Figures 5A–O). Across all groups, the dominant CAZy categories included Glycoside Hydrolases (GH) and Glycosyl Transferases (GT), followed by Carbohydrate Esterases (CE), whereas Polysaccharide Lyases (PL), Auxiliary Activities (AA), and Carbohydrate-Binding Modules (CBM) appeared at lower frequencies. GH families consistently represented the largest fraction of annotated CAZy enzymes, highlighting the central role of carbohydrate degradation in *L. reuteri* metabolism.

Rodent-derived strains possessed the most extensive CAZyme repertoire, particularly in GH and GT families. GH13, GH43, GH3, and GH36 were predominant, indicating a strong capacity for degrading diverse plant-derived polysaccharides and host mucins. Several GH families (e.g., GH31, GH32) and CE families were uniquely enriched, suggesting metabolic versatility in the complex gut environment of rodents. Human intestinal and fermented food isolates also showed a rich GH and GT repertoire, reflecting a strong adaptation to carbohydrate-rich environments. Fermented food strains shared similar CAZyme reductions with dairy isolates but maintained slightly higher counts of GT2, GT4, and GH73, indicating preservation of cell-wall modification and biofilm-forming capabilities. Their limited GH diversity suggests long-term adaptation to stable, nutrient-sufficient fermentation environments. Human intestinal isolates displayed intermediate CAZyme abundance, dominated by GH13, GH3, and GT2 families, reflecting a generalist carbohydrate-utilization strategy. The presence of several host-glycan-degrading enzymes (GH29, GH95) suggests adaptation to mucin utilization in the human gut. Dairy isolates exhibited marked genome contraction in CAZyme content, particularly within GH and CE families. GH13 and GH25 were the most conserved families, primarily associated with starch and peptidoglycan hydrolysis, respectively. The absence or reduction of hemicellulase-related GH43 and GH51families supports a pattern of functional specialization toward milk carbohydrate metabolism.

By contrast, bird-derived isolates and the “Others” group displayed comparatively lower representation across most CAZy categories. Isolates from ruminants displayed intermediate enzyme profiles, with moderately elevated GT and CE abundances, whereas dairy product isolates showed a balanced GH/GT distribution but low PL and AA content. CBM modules remained uniformly scarce across all groups.

These findings highlight the diversity and host-specificity of CAZymes in *L. reuteri* and emphasize that CAZy functional composition in *L. reuteri* is shaped by the ecological niche and host environment, reflecting a pattern of host-driven evolutionary adaptation. This plasticity in carbohydrate-active enzyme content likely contributes to its successful colonization of diverse vertebrate hosts and food matrices.

### 3.6 Probiotic-related gene annotation

**Figure 6:**
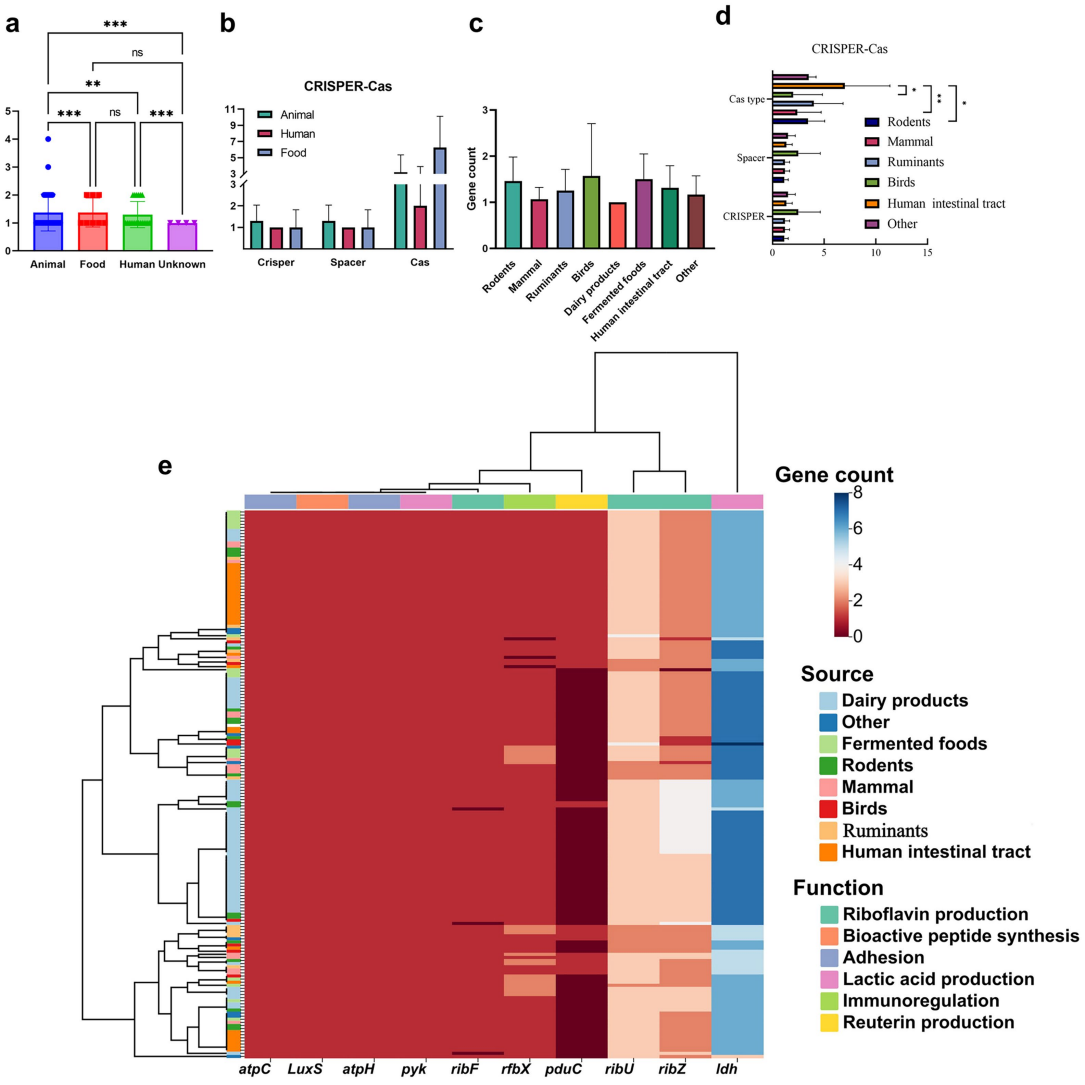
Other functional genomic features of *Limosilactobacillus reuteri* genomes from different sources. **(a,c)** Secondary metabolic gene clusters. Significant differences are indicated (**p < 0.01 and ***p < 0.001); ns denotes non-significant. **(b,d)** CRISPR-Cas system annotation and **(e)** probiotic function-related genes, including riboflavin production (*rib*F, *rib*X, *rib*U, *rib*Z), bioactive peptide synthesis (*lux*S), adhesion (*atp*C, *atp*H), lactic acid production (*py*K, ldh), immunoregulation (*rfb*X), and reuterin production (*pdu*C).

The *L. reuteri* genomes from different sources were annotated for probiotic-related genes (Figure 6e). Annotation of functional genes revealed widespread presence of *rib*F, *rib*X, *rib*U, and *rib*Z (riboflavin biosynthesis), *lux*S (bioactive peptide synthesis), *atp*C and *atp*H (adhesion), *py*K and ldh (lactic acid production), *rfb*X (immunomodulation), and *pdu*C (reuterin production). While all fractions contained these genes, their relative abundance varied, with some sources enriched in adhesion and metabolism-related functions. These findings align with known probiotic mechanisms in *L. reuteri* (Spinler et al., 2008; Frese et al., 2024; Yu et al., 2018). Rodents and Mammals host-associated groups showed enrichment for *luxS* and *pdu*C, together with detectable riboflavin genes (*rib*U, *rib*Z) in subsets. Ruminants profiles emphasized riboflavin-pathway genes with moderate *lux*S and *pdu*C, consistent with versatile nutrient metabolism. Birds isolates carried narrower panels, with comparatively fewer *lux*S and *pdu*C positives. Dairy products genomes were consistently high for glycolysis markers (*py*k, ldh) while showing reduced *lux*S/*pdu*C and patchy riboflavin genes. Fermented foods similar to dairy but with slightly broader riboflavin gene presence in a subset, consistent with multiple introductions into food chains.

Among human, animal, and food-derived, there were no significant differences (*p* > 0.05) among riboflavin-generating genes (*rib*F*, rib*Z, *rib*U), bioactive peptide genes (*Lux*S), adhesion genes (*atp*C, *atp*H), and lactic acid-producing genes (*pyk*). However, there were significant differences in the lactic acid-producing gene ldh, the immunomodulatory gene *rfb*X, and the encodes reuterin gene *pdu*C (*p* < 0.05). One-way ANOVA followed by Tukey’s HSD showed that human- and animal-derived (host-associated) isolates had higher detection frequencies of *pduC* and *rfb*X than dairy and fermented-food isolates (Tukey-adjusted *p* = 0.004–0.039), while *ldh* remained broadly conserved but reached higher counts in dairy genomes because of glycolysis specialization. The ldh gene encodes lactate dehydrogenase, which catalyzes the conversion of pyruvic acid to lactic acid and is the core gene for acid production in lactic acid bacteria (Nikkhah et al., 2024). Isolates may regulate acid production capacity through differences in ldh expression levels due to different pH requirements of the environment they are in, such as the human intestine or fermented foods, thereby inhibiting competing microorganisms (Hul et al., 2024). *rfb*X is involved in the synthesis of O antigen in lipopolysaccharide (LPS) of bacterial cell walls and affects the diversity of surface antigens. The differences in the recognition of O antigen by the immune systems of different hosts (humans, animals, and foods) may lead to other natural selection pressures of the *rfb*X gene (Klee et al., 2020).

The *pdu*C gene, responsible for the production of the antimicrobial compound reuterin, showed significant variation among isolation sources. The *pdu*C gene is significantly more prevalent in human- and animal-derived isolates compared to those from food sources. The reason for this might be because the *pdu*C gene may be involved in specific metabolic pathways (such as 1, 2-propanediol utilization), which play a key role in the colonization or competition of bacteria in the intestinal environment of human or animal hosts. For example, specific metabolic substrates may exist in the host’s intestine, promoting the selective retention of strains carrying the *pdu*C gene. However, food source isolates may not retain the gene due to the lack of relevant substrates in the environment or different metabolic requirements (Li et al., 2024). This aligns with the ability of many human intestinal and animal-derived *L. reuteri* to produce reuterin, a well-known antibacterial compound (Schaefer et al., 2010). It exhibits broad-spectrum antimicrobial activity, particularly against *L. reuteri*.

### 3.7 Drug resistance genes

Comparative genomic analysis was used to assess the safety of *L. reuteri* at the genomic level by identifying genes related to drug resistance, pathogenicity, and environmental adaptation. This approach provides valuable insights into the biosafety of *L. reuteri* as a probiotic (Plavec and Berlec, 2020). Strain-specific safety assessments are necessary prior to clinical or food applications.

Analysis of 176 *L. reuteri* strains revealed the presence of drug resistance genes in different derived isolates. A total of 17 species were annotated in this study, with animal-derived *L. reuteri* being the most abundant and food-derived being the least abundant (Supplementary Table S3). At the source level (ARG-positive genomes only), Mammal contributed the most hits, followed by Ruminants, birds, human intestinal tract, dairy products, rodents, and fermented foods. Functionally, tetracycline resistance genes *tet*(W/N/W), *tet*(W), *tet*(O/W), *tet*(O/W/O) dominated the dataset across sources, primarily *erm*(B) and lincosamide genes (*lnu*A) also present. A smaller number of chloramphenicol acetyltransferases and streptogramin A acetyltransferases (*vat*E) were detected.

Notably, the *tet* and *vatE* were identified in human intestinal-derived isolates SD2112 and SD-LREI-IT. *ErmB* was mainly detected in the food-derived HC5-4. Additionally, the lincomycin resistance gene *lnuA* was found in genomes from all isolation sources (animal, human, and food). Lincomycin, a lincosamide antibiotic, is widely used in livestock to treat respiratory and gastrointestinal infections. Its extensive use has contributed to the emergence and spread of resistance genes, with *Campylobacter* being a major global cause of bacterial foodborne diseases. An interesting finding is the presence of the *fex*A gene, which confers resistance to florfenicol, in the animal-derived isolate AN417. This gene has been previously identified in chicken feces and *Campylobacter jejuni* isolates from chickens. It was first reported on *Campylobacter* plasmids, providing evidence of horizontal gene transfer between Gram-positive and Gram-negative bacteria (Trieu-Cuot et al., 1985). Further studies indicate that *fex*A is located on a resistance gene island *tet(*L*)-fex*A*-cat*A*-tet(*O*)* and can be horizontally transferred, enhancing the adaptability of *Campylobacter jejuni* under florfenicol selection pressure (Zheng B. et al., 2015a).

### 3.8 Bacteriocins

Bacteriocins are antimicrobial peptides produced by bacteria that inhibit the growth of other microorganisms (Breukink et al., 1999). Unlike conventional antibiotics, bacteriocins are proteinaceous and typically exhibit narrow-spectrum activity, targeting strains of the same or closely related species. They are also associated with minimal resistance development, as spontaneous mutations conferring resistance are rare (Soltani et al., 2021). When resistance does occur, it is often due to modifications in the cell envelope, such as alterations in charge or thickness (Sakayori et al., 2003; McBride and Sonenshein, 2011).

Bacteriocin prediction analysis across *L. reuteri* isolates from different sources revealed that animal-derived genomes had the highest number of predicted bacteriocin gene clusters, while food-derived strains had the fewest (*p* < 0.001; Figure 6a). This may reflect a stronger antimicrobial and probiotic activity of *L. reuteri* isolates from animal sources (Hernandez-Gonzalez et al., 2021).

Host-associated groups (human, rodents, other mammals) collectively carry the majority of predicted bacteriocin loci, whereas food-origin genomes (dairy, fermented foods) harbor fewer, with dairy showing the lowest count (Figure 6c). Two main gene clusters encoding bacteriocins were identified in *L. reuteri* genomes (Supplementary Table S4), including enterolysin A: a class III bacteriocin originally purified from *Enterococcus fecalis* LMG Some studies have also shown that the lack of sufficient 2,333. It exhibits bacteriolytic activity against selected strains of *Enterococcus*, *Micrococcus*, *Lactococcus,* and *Lactobacillus*, with a dose-dependent killing effect (Nilsen et al., 2003). Sactipeptides: a class of ribosomally synthesized and post-translationally modified peptides characterized by sulfur-to-alpha carbon thioether cross-links. Sactipeptides display a broad spectrum of biological activities, including antibacterial, spermicidal, and hemolytic properties (Chen et al., 2021).

*Enterolysin*_A like loci are broadly distributed but are most frequent in human, rodent, and mammal isolates, mirroring the overall source ranking. Sactipeptides occur as minor, scattered hits across mammalian sources; they are rare or absent in dairy. Across sources, genomes carried 1–2 BGCs per strain with modest between-group dispersion. Human intestinal tract and fermented-food isolates showed slightly higher average BGC counts, whereas dairy and bird isolates tended to be lower. Rodents and mammal groups were intermediate with wider variance, consistent with mixed ecological pressures on specialized-metabolite capacity. These findings suggest that *L. reuteri* has the potential to produce bacteriocins, which could contribute to its probiotic and antimicrobial properties. The higher bacteriocin-cluster loads in host-associated sources are in line with their richer probiotic-gene repertoires and with the greater ARG diversity observed in some animal-derived isolates, pointing to stronger competitive and defensive pressures in animal and human intestinal niches.

### 3.9 CRISPR-Cas system

The CRISPR-Cas system is an adaptive immune system in prokaryotes that protects against the invasion of exogenous genetic elements, such as phages and plasmids (Xu and Li, 2020). This system consists of CRISPR arrays, which store fragments of foreign DNA, and Cas (CRISPR-associated) genes, which encode proteins responsible for targeting and cleaving invading DNA. Together, they form a highly conserved defense mechanism in bacteria.

In this study, the CRISPR-Cas systems of 176 *L. reuteri* isolates were annotated using online tools (Supplementary Table S5). While differences were observed in CRISPR arrays, Cas gene cluster types, and spacer numbers among genomes from animal, human intestinal tract, food isolation sources, these differences were not statistically significant (Figure 6b). However, after carefully classifying the sources of separation, counts of CRISPR arrays, spacers, and Cas-type diversity varied by the specific source (Figure 6d). Human intestinal tract isolates displayed the highest Cas-type richness and spacer loads, with significant pairwise differences from dairy and fermented-food cohorts. Rodents and mammal genomes showed intermediate CRISPR diversity, while food-origin (dairy food and fermented food) and bird groups were generally lower and more variable. CRISPR array length and spacer number varied across sources, indicating differences in adaptive immune capacity. Fractions with longer CRISPR arrays and higher spacer counts may reflect historical exposure to a broader spectrum of mobile genetic elements such as bacteriophages and plasmids (McCarty et al., 2020). Of the 176 strains, only 41 (23.3%) contained CRISPR-Cas systems, with the following distribution: 66% from animals, 20% from food sources, 12% from humans, and 2% from unknown origins. This suggests that animal-derived *L. reuteri* isolates are more likely to possess this defense mechanism, potentially reflecting stronger selective pressure from phages in animal hosts. The low prevalence of CRISPR-Cas systems in *L. reuteri* aligns with previous reports. While CRISPR-Cas systems are present in 85.2% of archaea and 42.3% of bacteria (Liu et al., 2023), only 17% of *L. reuteri* strains have been reported to contain these systems (Plagens et al., 2015). The relatively low occurrence of CRISPR-Cas systems in *L. reuteri* is consistent with its niche-specific evolutionary adaptations.

Notably, the *L. reuteri* genome contains a rich diversity of Type II-A CRISPR-Cas systems, which are known for their strong anti-phage activity. This further supports the hypothesis that animal-derived *L. reuteri* strains have evolved robust defense mechanisms against phage predation (Ksiezarek et al., 2022). The results of this study are consistent with findings in other lactic acid bacteria. For example, Crawley et al. reported that some bacterial species contain multiple CRISPR-Cas systems, while others, such as *Lactobacillus acidophilus*, encode CRISPR arrays but lack associated *cas* genes (Crawley et al., 2018). This parallels the observations in *L. reuteri*, where CRISPR-Cas systems are present in only a subset of isolates, highlighting the variability in CRISPR-Cas distribution across bacterial species. Together with the source-enriched bacteriocin clusters and the uneven ARG profiles, the CRISPR-Cas patterns support a unified view that ecological origin not only shapes core genome architecture but also modulates accessory, defense, and probiotic traits in *L. reuteri*.

## 4 Discussion

*Limosilactobacillus reuteri* is a Gram-positive probiotic that has garnered extensive attention from the industry and scientific community due to its high economic and application value. Previous studies have sequenced *L. reuteri* from herbivores. Evaluate genetic diversity and gain insight into the distinctive features (Zhang et al., 2024). However, little is known about the genomic diversity in their different niches. In this study, the whole-genome sequences of 176 *L. reuteri* strains from animal, human intestinal, and food sources were analyzed using comparative genomics. The genome characteristics of isolates obtained from eight ecological sources, including dairy products, fermented foods, rodents, mammals, ruminants, birds, the human intestinal tract, and miscellaneous environments were compared.

This study demonstrates a clear ecological signature in the genome evolution of *L. reuteri*. The genome reduction observed in dairy and fermentation-associated strains is consistent with niche specialization via reductive evolution. Genetically streamlined genomes are a common feature among lactic acid bacteria adapted to nutrient-rich and controlled environments such as milk or fermented foods, where metabolic flexibility becomes less essential for survival (Somerville et al., 2024; McAuliffe et al., 2019). Similar evolutionary patterns have been reported in *Lactococcus lactis* (Wels et al., 2019), *Streptococcus thermophilus* (Voula et al., 2019), and domesticated *Lactobacillus delbrueckii* strains (Baek et al., 2023). The loss of stress response and carbohydrate utilization genes during environmental adaptation has been widely documented and likely explains the smaller genomes observed in dairy isolates (Roux et al., 2022).

At an ANI threshold of ≥97%, the data were partitioned into 14 connected clusters, comprising 10 multi-member principal clusters and 4 single-member clusters. This pattern of 10 principal clusters, plus several single members, aligns in both magnitude and clarity with the 10 major lineages of *L. reuteri* reported by Li et al. (2023). That is, the ANI-based structure in this study reproduces the previously established intra-species hierarchical differentiation.

The blocky topology shows source as the primary axis of population structure in *L. reuteri*. Rodent and mammalian cohorts form the backbone, with human isolates were embedded. This is consistent with previous genomic-based evidence that host ecology shapes population structure with episodic cross-host transfers (Nayfach et al., 2021; Zheng et al., 2020). The compact, high-ANI dairy and fermentation clusters placement within mammalian sectors are hallmarks of domestication in food environments. Such patterns are well documented in lactic acid bacteria, including recent reconstructions of domestication patterns in *Streptococcus thermophilus* (Meyer and Jordi, 1987). The distinct bird block with sharper boundaries is compatible with avian-specific adaptation, paralleling reports of host-restricted *L. reuteri* lineages in birds (Li et al., 2023). ANI-tight food clusters align with genome streamlining and functional contraction whereas broader host clusters coincide with expanded DNA-repair, amino-acid and glycan metabolism typical of complex gut niches. These trends consistent with recent taxonomic and ecological syntheses for lactobacilli and gut microbes (Mejia-Caballero and Marco, 2025).

Consistent with prior population-genetic and experimental work (Gangaiah et al., 2024). Our phylogeny reproduces the robust source-associated clustering and hallmarks of host specialization reported previously. Rodent-associated clades occupy basal positions in our tree, consistent with deep animal associations; however, documented cross-host transfers argue for an animal-proximal ancestry rather than a single definitive animal origin. The aggregation of food-derived isolates is consistent with an intestinal ancestry, followed by matrix-associated selection in food environments, resulting in tight clusters nested within broader host-adapted lineages.

The ecological variation in COG functional profiles highlights distinct evolutionary strategies and functional adaptations of *L. reuteri*. The enrichment of L category genes in rodent- and bird-derived strains suggests that host-associated isolates maintain higher genome plasticity, likely as a strategy to adapt to dynamic gastrointestinal environments that exert strong selective and mutational pressures (Zhang et al., 2024).

In contrast, strains from dairy and fermented food niches exhibited marked gene reduction in categories L, E, R, and S, supporting a pattern of genome streamlining driven by ecological specialization. Reductive evolution is a common feature of domesticated lactic acid bacteria exposed to stable, nutrient-rich environments such as milk and fermented substrates (Somerville et al., 2024). The reduced number of E category genes suggests that food-origin strains rely on exogenous peptides and amino acids abundant in milk rather than maintaining complex amino acid biosynthetic pathways (Canon et al., 2022). Similarly, the significant reduction of R and S category gene counts in dairy isolates indicates loss of accessory genes and hypothetical functions, consistent with evolutionary simplification during long-term adaptation to fermentation environments (Somerville et al., 2024). In contrast, the larger R and S repertoires in host isolates likely reflect exposure to genetic exchange within complex gut microbiomes, leading to the acquisition of mobile genetic elements and niche-specific functions (Schubert et al., 2025; Tang et al., 2025).

Previous studies have found that *L. reuteri* is rich in the CAZymes family (Dong et al., 2024). In this study, this view was further proved. At the same time, it was found that there were significant differences in the CAZymes family of *L. reuteri* from different sources, especially the GHs and GTs families, which may be related to the utilization of carbon sources. The elevated GH abundance in rodent isolates likely reflects adaptation to the high-fiber diets of rodent hosts, where efficient carbohydrate degradation provides a competitive advantage (Yu et al., 2018). Similarly, the rich CAZy profile of human intestinal and fermented food strains may support flexible nutrient utilization in environments with complex and diverse carbohydrate sources.

Conversely, the lower CAZy diversity observed in bird isolates and the “Others” group may indicate niche specialization or reduced dependence on complex polysaccharide metabolism. The moderate enrichment of CE families in herbivore isolates suggests enhanced ability for modification of plant-derived polysaccharides, supporting adaptation to ruminants (La Rosa et al., 2022). The uniformly low abundance of PL, AA, and CBM modules indicates that *L. reuteri* is not a primary degrader of recalcitrant plant cell wall polysaccharides, unlike members of genera such as *Bacteroides* or *Ruminococcus* (Kim et al., 2024). Instead, its functional strategy appears to rely on utilizing more accessible carbohydrates or metabolic cooperation with other community members.

It is worth noting that the food source GH73, GH25, and GH13 families are particularly abundant. These families are involved in the degradation of hard-to-digest carbohydrates in staple foods and dairy products. Notably, GH13 was particularly abundant in food-source isolates and is classically associated with the metabolism of starch and other *α*-glucans. By contrast, GH25 and GH73 encode peptidoglycan hydrolases implicated in cell-wall remodeling and autolysis rather than dietary carbohydrate breakdown. However, by elucidating source-specific differences, we discovered that animal-derived strains exhibit greater diversity of carbohydrate-active enzymes, increased capacity for antibiotic production, more antimicrobial resistance genes, and stronger phage resistance. These characteristics correlate with complex herbivorous or omnivorous dietary structures and intense interspecies interactions (Bredon et al., 2020). Conversely, food isolates enrich GH13 and cell wall hydrolases (GH25/GH73). Food-derived strains, especially those from fermented food sources, are unique in the type and quantity of carbohydrate-active enzymes they produce. GT27 was found only in food-derived isolates, and to our knowledge, this is the first comparative genomic evidence that a specific O-glycan-initiating glycosyltransferase is a marker of food-adapted *L. reuteri* lineage. This suggests that long-term colonization in dairy products and fermentation substrates has led this strain to evolve a unique carbohydrate processing module that is not present in host-associated strains. CAZy functional composition in *L. reuteri* is shaped by the ecological niche and host environment, reflecting a pattern of host-driven evolutionary adaptation. This plasticity in carbohydrate-active enzyme content likely contributes to its successful colonization of diverse vertebrate hosts and food matrices.

Antibiotics are widely used as feed additives to promote growth or prevent diseases in animal breeding, such as tetracyclines and macrolides, which are subject to prolonged and intensive use (Mohammed et al., 2022). The results of drug resistance annotation for different isolates showed that the most common drug resistance genes included the tetracycline resistance gene (*tet*), the clindamycin resistance gene (*erm*B), and the glycopeptide resistance gene (*vat*E). Mammalian strains have the highest resistance among animal-derived isolates. The food source isolates were the least. These in silico findings indicate that genetic potential, phenotypic resistance, and mobility require laboratory verification.

The widespread use of antibiotics leads to selection pressure, promoting the production of drug-resistant strains and genes. The environment in animals is conducive to the horizontal transfer of drug resistance genes; food-borne isolates may have fewer drug resistance genes due to processing and lack of continuous antibiotic exposure. The long-term use of tetracycline in chicken farming has led to almost all Campylobacter isolates carrying the *tet* (O) gene (Wang et al., 2016). This continuous selection pressure promotes the enrichment of genes conferring drug resistance in the animal intestinal flora. Bacteriophages are often used as substitutes for antibiotics to control pathogens (Park et al., 2025). The inconsistency of the phage application effect may reflect that the strain has acquired resistance through adaptive evolution. Human intestinal flora is relatively less affected by phage pressure, and the use of antibiotics is primarily aimed at acute infections rather than long-term prevention, resulting in low pressure for the evolution of resistance (Rohde et al., 2018). The extensive use of antibiotics in animal breeding has prompted the development of strains that produce multi-drug resistance mechanisms, which may affect their resistance to phages. In this case, due to exposure to different selection pressures, strains from different sources may exhibit different resistance gene profiles. Therefore, the use of animal-derived strains requires greater caution. Whether the drug resistance gene is expressed still needs further *in vitro* experiments to verify.

In terms of probiotic function, genes involved in riboflavin production (*rib*F), bioactive peptide synthesis (*Lux*S), adhesion genes (*atp*C, *atp*H), and lactic acid production (*py*K) were mainly annotated. In particular, *Pdu*C, which only exists in *L. reuteri*, is involved in the synthesis of the broad-spectrum antimicrobial substance reuterin. This substance inhibits the growth of pathogenic bacteria, such as *Staphylococcus aureus*, and enhances the strain’s tolerance to the gastrointestinal environment (Viladomiu et al., 2021). The *pdu*C gene was 48.1% of animal isolates and 65.7% of human intestinal isolates. Food-borne isolates only contained 15.3% of the gene. In specific Ruminants, *pdu*C abundance was higher; this may relate to substrate availability in those gut environments. Such ecological explanations remain tentative pending targeted measurements. However, the environment in which food-derived microorganisms are located (such as oxygen and nutritional conditions) may lack the selective pressure for the continuous use of propylene glycol. For example, bacteria in food are more dependent on other carbon sources, such as glucose, without the need to metabolize propylene glycol through *pdu*C (Tao et al., 2019). Some studies have also shown that the lack of sufficient nutrients in the environment where foodborne *L. reuteri* is located may lead to the inhibition of the expression of its *pdu*C gene (Viladomiu et al., 2017), thereby reducing the *pdu*C content. This may be the reason for the low *pdu*C amount of food-borne *L. reuteri.* This niche isolation pattern of the *pdu*C reuterin pathway provides a genomic explanation for previously observed differences in reuterin production capacity among *L. reuteri* sources and represents a novel ecological signal discovered in this dataset.

Host-adapted lineages, such as rodents, mammals, herbivores, and humans, possess diverse CRISPR-Cas defense mechanisms and probiotic functional modules (*lux*S, *pdu*C, and riboflavin genes), enabling them to survive in the phage-rich, immune-active gut. Food-related lineages, such as those associated with dairy and fermented foods, exhibit lower CRISPR diversity and simplified functionality, which facilitates rapid acid production and enhances the stability of stable fermentation ecosystems. Given sampling biases and in silico annotation limits, phenotypic validation and broader, balanced sampling will be essential in future work.

Although this study provides a comprehensive in silico overview of carbohydrate-active enzymes, bacteriocin gene clusters, CRISPR-Cas systems, and antibiotic resistance genes in *L. reuteri*, these predictions do not by themselves demonstrate phenotypic functionality. Therefore, future research should include wet-lab validation. For example, the presence of the *pdu*C gene in host-associated strains suggests their potential to produce reuterin, but this needs to be confirmed through gene expression analysis and biochemical analysis of reuterin yield (Bortolaia et al., 2020). Similarly, antibiotic resistance genes (ARGs) detected by ResFinder need to be validated through phenotypic susceptibility testing to determine whether the predicted determinants are expressed and confer resistance to these strains (Feldgarden et al., 2019). This combined genome-phenotype assessment is crucial for supporting strain-level safety assessments and screening isolates with industrial application value.

From an application perspective, these niche-linked genomic signatures provide a rational basis for pre-selecting *L. reuteri* strains. Host-derived isolates that combine *pdu*C, riboflavin and *lux*S modules together with diversified CRISPR-Cas systems are promising candidates for gut-targeted probiotic development, but their relatively higher ARG carriage means they should undergo strict phenotypic susceptibility testing before use. Conversely, food-derived isolates that show genome streamlining, rich CAZy content, and low ARG content are better suited for stable dairy or fermented-food starter cultures, where rapid acidification and predictable performance are prioritized over broad antimicrobial activity. Thus, the comparative-genomic framework presented here can directly inform strain-level safety assessment and application matching.

## 5 Conclusion

Analyzing 176 *Limosilactobacillus reuteri* genomes, we identified significant variations in features like the core/pan-genome, CAZymes, bacteriocin production, CRISPR-Cas systems, and antibiotic resistance genes. The core-gene phylogeny identified seven clusters and revealed that isolation rather than geography is the primary driver of divergence. Mammal strains have broader functional repertoires and stronger anti-phage defenses, whereas human intestinal tract and dairy products strains have niche-specific adaptations. The functional composition of *L. reuteri* is influenced by its different ecological niches and host environments, reflecting a host-driven evolutionary adaptation pattern. Collectively, these patterns highlight environment-specific genomic architectures and reinforce the use of *L. reuteri* as a versatile and safe probiotic for strain-specific applications in the food, health, and biotechnology industries.

## Data Availability

The data that support the findings of this study have been deposited into National Genomics Data Center (Ma et al., 2025), Beijing Institute of Genomics (Members C.-N. and Partners, 2025), Chinese Academy of Sciences/China National Center for Bioinformation, under accession number PRJCA042243 that is publicly accessible at https://ngdc.cncb.ac.cn/gwh.

## Footnotes

1
http://sourceforge.net/proiects/soapdenovo2/files/GapCloser/

2
http://eggnog-mapper.embl.de/

3
https://itol.embl.de/software

4
http://csb1.bmb.uga.edu/dbCAN/

5
http://www.cazy.org/

6
https://cge.food.dtu.dk/ser-vices/ResFinder-4.1/

7
https://cge.food.dtu.dk/ser-vices/Virulence/

8
https://www.mgc.ac.cn/cgi-bin/VFs/v5/main.cgi
