# Supplementary material for: Genomic diversity and functional adaptation of Limosilactobacillus reuteri isolated from diverse ecological niches
Source: Front Microbiol. 2025 Dec 12;16:1732127. doi: 10.3389/fmicb.2025.1732127 (PMC12741103; doi:10.3389/fmicb.2025.1732127)
Supplement: Supplementary file 1 [file Table_1.docx]

Supplementary Material

# Supplementary Figures and Tables

## Supplementary Figures


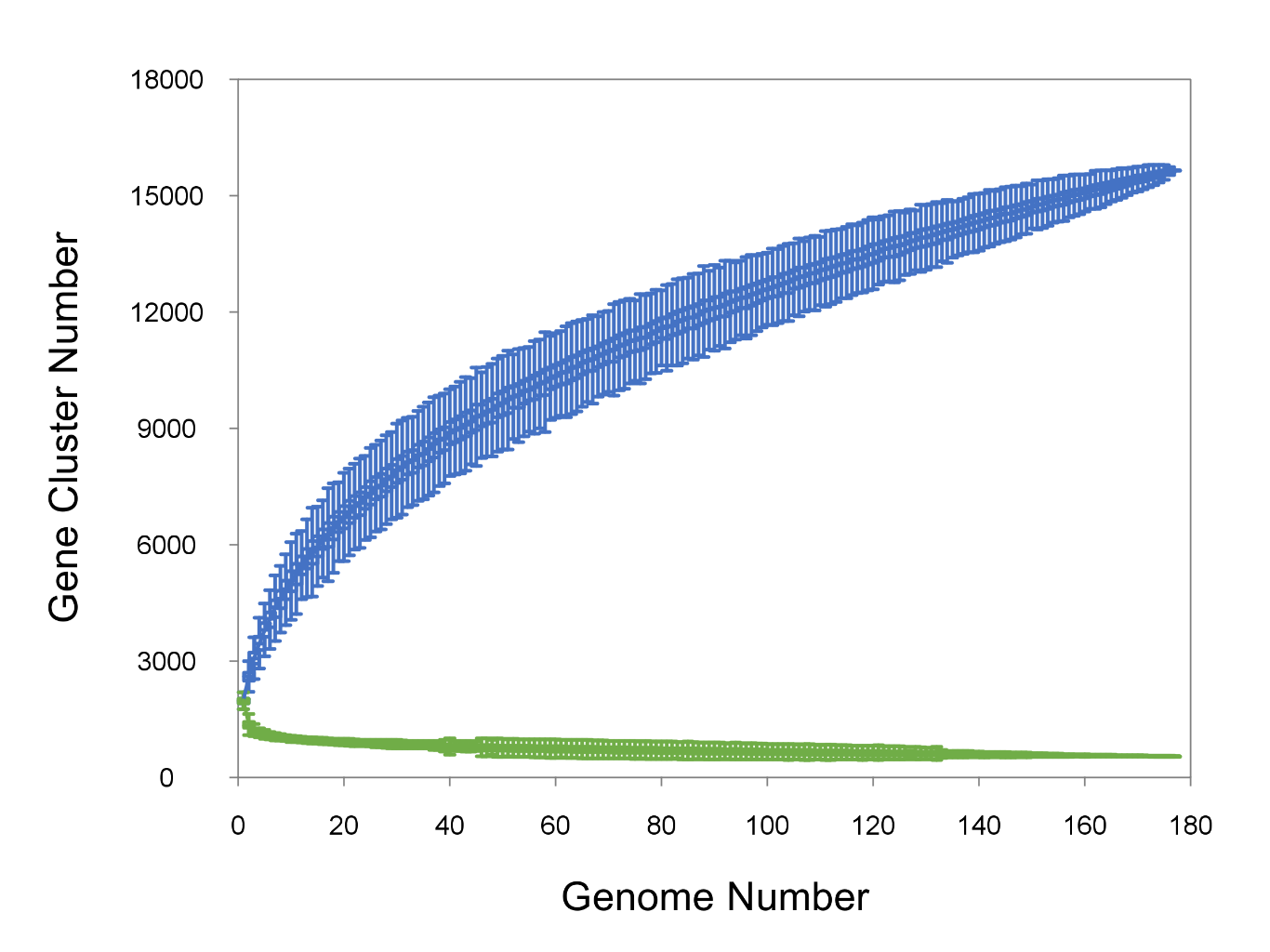


**Supplementary Figure S1.**  **Accumulation curves for the pan-genome and core-genome of the species *Limosilactobacillus reuteri***

**
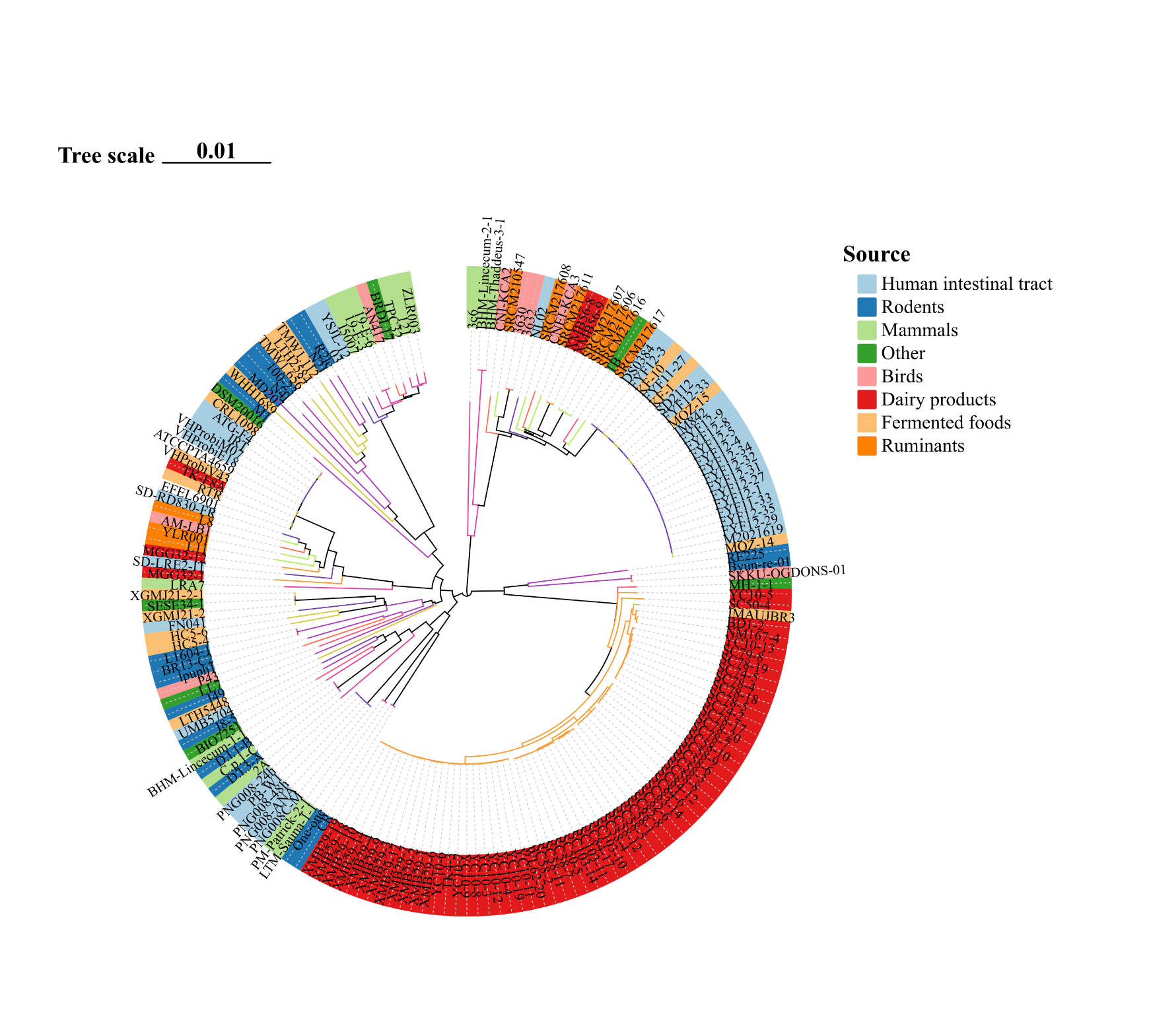
**

**Supplementary Figure S2. Core-gene phylogenetic tree of 176 *Limosilactobacillus reuteri* genomes. The phylogenetic tree was reconstructed with Neighbor-Joining method, based on 553 core genes shared across all *Limosilactobacillus reuteri* genomes in the dataset, showing genetic divergence (tree scale) and evolutionary relationships. Outer rings indicate isolation source, respectively, highlighting links between phylogeny, distribution, and ecology.**

**1.2 Supplementary Tables**

**Table S1. Habitats, sources, and regions of 181 *Limosilactobacillus reuteri* genomes**

| **Genome ID** | **Habitat** | **Habitat** | **Source** | **Region** | | **Accession number** |
| --- | --- | --- | --- | --- | --- | --- |
| 03 | Human | Human intestinal tract | Human feces | China | GCF_030517815.1 (NCBI) | |
| 100-23 | Animal | Rodents | Rodent | America | GCF_000168255.1 (NCBI) | |
| 121 | Animal | Mammal | Pig | Holland | GCF_001889975.1 (NCBI) | |
| 19-E-3 | Animal | Mammal | Wild boar colon | America | GCA_020412485.1 (NCBI) | |
| 19-E-6 | Animal | Mammal | Wild boar colon | America | GCF_020412465.1 (NCBI) | |
| 1B | Animal | Other | *Equus caballus* | Australia | GCA_013487925.1 (NCBI) | |
| 2A | Animal | Mammal | Porcupine | China | GCA_020784255.1 (NCBI) | |
| 3630 | Animal | Birds | Chicken cecum | Germany | GCF_020978225.1 (NCBI) | |
| 3632 | Animal | Birds | Chicken | Germany | GCF_020978285.1 (NCBI) | |
| 3c6 | Animal | Mammal | Swine manure | Britain | GCA_036621895.1 (NCBI) | |
| AM-LB1 | Animal | Birds | Chicken manure | Russia | GCA_025369755.1 (NCBI) | |
| AN417 | Animal | Birds | Poultry | Korea | GCF_013348825.1 (NCBI) | |
| ATCC PTA 4659 | Unknown | Other | Unknown | unknown | GCF_030418275.1 (NCBI) | |
| ATG-F4 | Human | Human intestinal tract | Korean | Korea | GCF_004208615.1 (NCBI) | |
| BD1-2 | Food | Dairy products | Golden milk | China |  | |
| BHM-Lincecum-1-1 | Animal | Mammal | Mono | China | GCA_020785115.1 (NCBI) | |
| BHM-Lincecum-2-1 | Animal | Mammal | Mono | China | GCF_020785135.1 (NCBI) | |
| BHM-Thaddeus-3-1 | Animal | Mammal | Mono | China | GCF_020784195.1 (NCBI) | |
| BIO7251 | Food | Other | *Corsica citrus* | France | GCF_030262475.1 (NCBI) | |
| BR13-C2 | Animal | Rodents | Neonatal mice | America | GCA_022511545.1 (NCBI) | |
| BRDL17 | Unknown | Other | Feces | Korea | GCA_026183435.1 (NCBI) | |
| Byun-re-01 | Animal | Rodents | Rodent duodenum | Korea | GCA_003316895.1 (NCBI) | |
| CC-AA2-2 | Animal | Rodents | *Apodemus agrarius* | China | GCA_020784665.1 (NCBI) | |
| CNEI-KCA3 | Animal | Birds | Chicken feces | Nigeria | GCF_013694365.1 (NCBI) | |
| CNI-KCA2 | Animal | Birds | Chicken feces | Nigeria | GCF_012275185.1 (NCBI) | |
| C.p.1-C | Animal | Mammal | Brazilian porcupine | China | GCF_020784575.1 (NCBI) | |
| CR | Animal | Rodents | Rat intestine | America | GCF_009649505.1 (NCBI) | |
| CRL1098 | Food | Fermented foods | Sourdough bread | Germany | GCF_001657495.1 (NCBI) | |
| D.l.1-B | Animal | Rodents | Squirrel | Germany | GCF_020784555.1 (NCBI) | |
| D.l.3-A | Animal | Rodents | Squirrel | Germany | GCA_020784635.1 (NCBI) | |
| DS0384 | Human | Human intestinal tract | Korean baby feces | Korea | GCF_021398615.1 (NCBI) | |
| DSM20016 | Unknown | Other | Unknown | unknown | GCA_001434615.1 (NCBI) | |
| EFEL6901 | Unknown | Other | Feces (unknown source) | Korea | GCA_023078415.1 (NCBI) | |
| FN041 | Human | Human intestinal tract | Breast milk | China | GCA_019336465.1 (NCBI) | |
| FYF11-27 | Human | Human intestinal tract | 2-Month-old baby feces | China |  | |
| FYF11-33 | Human | Human intestinal tract | 2-Month-old baby feces | China |  | |
| FYF12-1 | Human | Human intestinal tract | Human feces | China |  | |
| FYF12-27 | Human | Human intestinal tract | Human feces | China |  | |
| FYF12-29 | Human | Human intestinal tract | Human feces | China |  | |
| FYF12-31 | Human | Human intestinal tract | Human feces | China |  | |
| FYF12-32 | Human | Human intestinal tract | Human feces | China |  | |
| FYF12-33 | Human | Human intestinal tract | Human feces | China |  | |
| FYF12-34 | Human | Human intestinal tract | Human feces | China |  | |
| FYF12-35 | Human | Human intestinal tract | Human feces | China |  | |
| FYF12-3 | Human | Human intestinal tract | Human feces | China |  | |
| FYF12-4 | Human | Human intestinal tract | Human feces | China |  | |
| FYF12-5 | Human | Human intestinal tract | Human feces | China |  | |
| FYF12-8 | Human | Human intestinal tract | Human feces | China |  | |
| FYF12-9 | Human | Human intestinal tract | Human feces | China |  | |
| HC5-4 | Food | Fermented foods | Rice wine | China |  | |
| HC5-6 | Food | Fermented foods | Rice wine | China |  | |
| I49 | Animal | Rodents | Rice wine | Switzerland | GCF_001688685.2 (NCBI) | |
| I5007 | Animal | Mammal | Healthy weaned piglets | China | GCF_000410995.1 (NCBI) | |
| I8-5 | Animal | Rodents | Rat | America | GCF_004684995.1 (NCBI) | |
| IM842 | Human | Human intestinal tract | Human breast milk | The Republic of Slovenia | GCF_925280345.1 (NCBI) | |
| IMAUJBR3 | Food | Fermented foods | Traditional air-dried mutton product | China | GCA_034259105.1 (NCBI) | |
| IRT | Human | Human intestinal tract | Human | Korea | GCA_001046835.1 (NCBI) | |
| KF-10 | Food | Fermented foods | Coffee | Arica |  | |
| KF-1 | Food | Fermented foods | Coffee | Arica |  | |
| L11 | Animal | Ruminants | Cattle colon | Canada | GCA_022642025.1 (NCBI) | |
| L1604-1 | Animal | Rodents | Rat intestine | America | GCF_009649545.1 (NCBI) | |
| L8 | Animal | Ruminants | Bovine rectum | Canada | GCF_022642805.1 (NCBI) | |
| LL7 | Unknown | Other | Gastric content | America | GCF_007633215.1 (NCBI) | |
| lpuph1 | Animal | Rodents | Rodent | America | GCF_000179455.1 (NCBI) | |
| Lr4000 | Animal | Rodents | Rat intestine | America | GCA_009649495.1 (NCBI) | |
| LRA7 | Animal | Mammal | Dog | China | GCA_036323925.1 (NCBI) | |
| LTH2584 | Food | Fermented foods | Sourdough | Germany | GCA_000712555.1 (NCBI) | |
| LTH5448 | Food | Fermented foods | Sourdough | Germany | GCF_000758185.1 (NCBI) | |
| LTM-Sauna-1-1 | Animal | Mammal | Lion-tailed macaque | China | GCF_020785015.1 (NCBI) | |
| M1 | Animal | Rodents | Mouse feces | China | GCA_030345055.1 (NCBI) | |
| M2021619 | Human | Human intestinal tract | Fecal samples of a healthy young woman | China | GCF_021459965.1 (NCBI) | |
| MD207 | Animal | Rodents | Mouse feces | France | GCA_010206285.1 (NCBI) | |
| MGG32-12 | Food | Dairy products | Fresh mare's milk | China |  | |
| MGG32-1 | Food | Dairy products | Fresh mare's milk | China |  | |
| MH-1-1 | Food | Other | Sour porridge | China |  | |
| MQZ-14 | Food | Fermented foods | Sourdough | Arica |  | |
| MQZ-15 | Food | Fermented foods | Sourdough | Arica |  | |
| N2J | Animal | Rodents | Rat intestine | America | GCA_009649475.1 (NCBI) | |
| N4I | Animal | Rodents | Rat | America | GCA_009649605.1 (NCBI) | |
| NL02 | Human | Human intestinal tract | Human | China | GCA_030585585.1 (NCBI) | |
| NM167-4 | Food | Dairy products | Sour milk | China |  | |
| One-one | Animal | Rodents | Rat intestine | America | GCA_009649535.1 (NCBI) | |
| P43 | Animal | Birds | Red bird | America | GCA_033570435.1 (NCBI) | |
| PB-W1 | Human | Human intestinal tract | Human | China | GCA_020785355.1 (NCBI) | |
| PM-Patrick-2-1 | Animal | Mammal | Guenon | China | GCA_020784895.1 (NCBI) | |
| PNG008-24h | Human | Human intestinal tract | Human | China | GCA_020785475.1 (NCBI) | |
| PNG008-48h | Human | Human intestinal tract | Human | China | GCA_020785415.1 (NCBI) | |
| PNG008-ANA | Human | Human intestinal tract | Human | China | GCF_020785335.1 (NCBI) | |
| PNG008C-M | Human | Human intestinal tract | Human | China | GCA_020785255.1 (NCBI) | |
| R2lc | Animal | Rodents | Rat | America | GCF_003703875.1 (NCBI) | |
| RE225 | Animal | Rodents | Rat gastrointestinal tract | China | GCF_030721705.1 (NCBI) | |
| RTR | Food | Fermented foods | Fermented fruit | Korea | GCF_021383585.1 (NCBI) | |
| SC103-10 | Food | Dairy products | Fresh yak milk | China |  | |
| SC103-11 | Food | Dairy products | Fresh yak milk | China |  | |
| SC103-14 | Food | Dairy products | Fresh yak milk | China |  | |
| SC50-4 | Food | Dairy products | Cheese | China |  | |
| SC50-5 | Food | Dairy products | Cheese | China |  | |
| SC58-10 | Food | Dairy products | Milk cake | China |  | |
| SC58-11 | Food | Dairy products | Milk cake | China |  | |
| SC58-12 | Food | Dairy products | Milk cake | China |  | |
| SC58-1 | Food | Dairy products | Milk cake | China |  | |
| SC58-2 | Food | Dairy products | Milk cake | China |  | |
| SC58-3 | Food | Dairy products | Milk cake | China |  | |
| SC58-4 | Food | Dairy products | Milk cake | China |  | |
| SC58-5 | Food | Dairy products | Milk cake | China |  | |
| SC58-6 | Food | Dairy products | Milk cake | China |  | |
| SC58-7 | Food | Dairy products | Milk cake | China |  | |
| SC58-8 | Food | Dairy products | Milk cake | China |  | |
| SC58-9 | Food | Dairy products | Milk cake | China |  | |
| SC74-2 | Food | Dairy products | Milk cake | China |  | |
| SC74-5 | Food | Dairy products | Milk cake | China |  | |
| SC78-17 | Food | Dairy products | Whey | China |  | |
| SC78-18 | Food | Dairy products | Whey | China |  | |
| SC78-19 | Food | Dairy products | Whey | China |  | |
| SC78-20 | Food | Dairy products | Whey | China |  | |
| SC78-4 | Food | Dairy products | Whey | China |  | |
| SC78-5 | Food | Dairy products | Whey | China |  | |
| SC78-7 | Food | Dairy products | Whey | China |  | |
| SC79-8 | Food | Dairy products | *Qula* | China |  | |
| SC82-6 | Food | Dairy products | Fresh yak milk | China |  | |
| SC82-8 | Food | Dairy products | Fresh yak milk | China |  | |
| SC83-14 | Food | Dairy products | Fresh yak milk | China |  | |
| SC84-12 | Food | Dairy products | Fermented yak milk | China |  | |
| SC84-17 | Food | Dairy products | Fermented yak milk | China |  | |
| SC84-6 | Food | Dairy products | Fermented yak milk | China |  | |
| SD2112 | Human | Human intestinal tract | Human | America | GCF_000159455.2 (NCBI) | |
| SD-LRE2-IT | Human | Human intestinal tract | Human | Italy | GCA_020023775.1 (NCBI) | |
| SD-RD830-FR | Human | Human intestinal tract | French | America | GCF_020023755.1 (NCBI) | |
| SESE34-1 | Food | Other | Sour porridge | China |  | |
| SKKU-OGDONS-01 | Animal | Birds | Chicken small intestine | unknown | GCF_003316935.1 (NCBI) | |
| SRCM210547 | Animal | Ruminants | Animal dropping | Korea | GCF_025515425.1 (NCBI) | |
| SRCM217606 | Animal | Ruminants | Bovine feces | Korea | GCA_028656265.1 (NCBI) | |
| SRCM217607 | Animal | Ruminants | Cattle | Korea | GCF_028656325.1 (NCBI) | |
| SRCM217608 | Animal | Ruminants | Bovine feces | Korea | GCF_028656315.1 (NCBI) | |
| SRCM217611 | Animal | Ruminants | Bovine feces | Korea | GCF_028656395.1 (NCBI) | |
| SRCM217616 | Animal | Ruminants | Bovine feces | Korea | GCF_028656195.1 (NCBI) | |
| SRCM217617 | Animal | Ruminants | Bovine feces | Korea | GCA_028656225.1 (NCBI) | |
| TK4-3 | Food | Dairy products | Sour milk | China |  | |
| TK-F8A | Food | Dairy products | Protein product | China | GCF_015377805.1 (NCBI) | |
| TMW1.112 | Food | Fermented foods | Sourdough | Germany | GCF_000722535.2 (NCBI) | |
| TMW1.656 | Food | Fermented foods | Sourdough | Germany | GCA_000712565.2 (NCBI) | |
| TPC32 | Animal | Mammal | Tibetan pig droppings | China | GCA_036903235.1 (NCBI) | |
| UMB5704 | Human | Human intestinal tract | Human | America | GCA_030226325.1 (NCBI) | |
| VA24-5 | Human | Human intestinal tract | Human vagina | Thailand | GCA_011028005.1 (NCBI) | |
| VHProbiE18 | Human | Human intestinal tract | Feces | China | GCF_021165875.1 (NCBI) | |
| VHProbiM07 | Human | Ruminants | Breast milk | China | GCF_021228055.1 (NCBI) | |
| VHProbiV43 | Food | Fermented foods | Pickle | China | GCF_035928175.1 (NCBI) | |
| WF-AA1-A | Animal | Rodents | *Apodemus agrarius* | China | GCF_020784695.1 (NCBI) | |
| WF-AA1-C | Animal | Rodents | *Apodemus agrarius* | China | GCA_020784725.1 (NCBI) | |
| WHH1689 | Food | Fermented foods | *Qingke* wine | China | GCA_003072625.1 (NCBI) | |
| WL62-20 | Food | Dairy products | Milk curd | China |  | |
| XGMJ21-2-1 | Food | Fermented foods | Rice wine | China |  | |
| XGMJ21-2 | Food | Fermented foods | Rice wine | China |  | |
| XMB29-10 | Food | Dairy products | milk curd | China |  | |
| XMB29-14 | Food | Dairy products | Milk curd | China |  | |
| XMB29-17 | Food | Dairy products | Milk curd | China |  | |
| XMB29-18 | Food | Dairy products | Milk curd | China |  | |
| XMB29-19 | Food | Dairy products | Milk curd | China |  | |
| XMB29-1 | Food | Dairy products | Milk curd | China |  | |
| XMB29-2 | Food | Dairy products | Milk curd | China |  | |
| XMB29-3 | Food | Dairy products | Milk curd | China |  | |
| XMB29-4 | Food | Dairy products | Milk curd | China |  | |
| XMB29-6 | Food | Dairy products | Milk curd | China |  | |
| XMB29-7 | Food | Dairy products | Milk curd | China |  | |
| XMB29-9 | Food | Dairy products | Milk curd | China |  | |
| XMB56-8 | Food | Dairy products | Fermented mare's milk | China |  | |
| XMB56-9 | Food | Dairy products | Fermented mare's milk | China |  | |
| YC10-11 | Food | Dairy products | Sour milk | China |  | |
| YC10-12 | Food | Dairy products | Sour milk | China |  | |
| YC10-13 | Food | Dairy products | Sour milk | China |  | |
| YC10-19 | Food | Dairy products | Sour milk | China |  | |
| YC10-3 | Food | Dairy products | Sour milk | China |  | |
| YC10-4 | Food | Dairy products | Sour milk | China |  | |
| YC10-5 | Food | Dairy products | Sour milk | China |  | |
| YC10-6 | Food | Dairy products | Sour milk | China |  | |
| YC10-7 | Food | Dairy products | Sour milk | China |  | |
| YC10-8 | Food | Dairy products | Sour milk | China |  | |
| YC10-9 | Food | Dairy products | Sour milk | China |  | |
| YLR001 | Animal | Ruminants | Yak | China | GCF_018884225.1 (NCBI) | |
| YSJL-12 | Human | Human intestinal tract | Fresh intestinal feces | China | GCF_006874665.1 (NCBI) | |
| ZLR003 | Animal | Mammal | Cecal mucosa of a healthy weaned piglet | China | GCA_001618905.1 (NCBI) | |

**Table S2. Features of 181 *Limosilactobacillus reuteri* genomes**

| **Genome ID** | **Length (bp)** | **GC Content (%)** | **Number of Coding Sequences** |
| --- | --- | --- | --- |
| 03 | 2255551 | 38.9 | 2225 |
| 100-23 | 2305557 | 38.73 | 2170 |
| 121 | 2302234 | 39.01 | 2210 |
| 19-E-3 | 1947745 | 38.97 | 1879 |
| 19-E-6 | 1947772 | 38.97 | 1876 |
| 1B | 2272714 | 38.93 | 2257 |
| 2A | 2202969 | 38.41 | 2083 |
| 3630 | 2399145 | 38.69 | 2467 |
| 3632 | 2483413 | 38.53 | 2595 |
| 3c6 | 1934800 | 38.68 | 1859 |
| AM-LB1 | 2346013 | 38.91 | 2328 |
| AN417 | 2162818 | 38.95 | 2104 |
| ATCCPTA4659 | 2096828 | 38.88 | 2111 |
| ATG-F4 | 2041516 | 38.89 | 2023 |
| BD1-2 | 1897512 | 38.69 | 1902 |
| BHM-Lincecum-1-1 | 2099280 | 38.63 | 1883 |
| BHM-Lincecum-2-1 | 2199526 | 38.36 | 2042 |
| BHM-Thaddeus-3-1 | 2278083 | 38.34 | 2151 |
| BIO7251 | 2100168 | 38.56 | 1919 |
| BR13-C2 | 2090281 | 38.29 | 1947 |
| BRDL17 | 1996211 | 38.98 | 1926 |
| Byun-re-01 | 2244514 | 38.88 | 2083 |
| CC-AA2-2 | 2130452 | 38.37 | 2024 |
| CNEI-KCA3 | 2085667 | 39.43 | 2404 |
| CNI-KCA2 | 2072001 | 38.92 | 2104 |
| C.p.1-C | 2187769 | 38.54 | 2040 |
| CR | 2171732 | 38.53 | 2036 |
| CRL1098 | 1963029 | 38.74 | 1942 |
| D.l.1-B | 2190901 | 38.56 | 2042 |
| D.l.3-A | 2175706 | 38.55 | 2027 |
| DS0384 | 2243237 | 39.04 | 2193 |
| DSM20016 | 1935860 | 38.63 | 1908 |
| EFEL6901 | 1972516 | 38.9 | 1971 |
| FN041 | 2391204 | 38.39 | 2298 |
| FYF11-27 | 1930551 | 38.64 | 1877 |
| FYF11-33 | 2067030 | 38.81 | 1948 |
| FYF12-1 | 1947981 | 38.68 | 1890 |
| FYF12-27 | 1987552 | 38.72 | 1932 |
| FYF12-29 | 2013224 | 38.75 | 1942 |
| FYF12-31 | 2001947 | 38.73 | 1940 |
| FYF12-32 | 2067789 | 38.8 | 1953 |
| FYF12-33 | 2015124 | 38.75 | 1954 |
| FYF12-34 | 2074972 | 38.81 | 1957 |
| FYF12-35 | 2020647 | 38.75 | 1951 |
| FYF12-3 | 1980958 | 38.73 | 1918 |
| FYF12-4 | 2065847 | 38.8 | 1949 |
| FYF12-5 | 2031627 | 38.73 | 1964 |
| FYF12-8 | 2068995 | 38.8 | 1951 |
| FYF12-9 | 2095444 | 38.79 | 1972 |
| HC5-4 | 2043645 | 38.85 | 1959 |
| HC5-6 | 1921151 | 38.6 | 1862 |
| I49 | 2044771 | 38.76 | 1894 |
| I5007 | 2093275 | 38.93 | 2032 |
| I8-5 | 2290627 | 38.3 | 2163 |
| IM842 | 2198312 | 38.94 | 2121 |
| IMAUJBR3 | 2272649 | 39.17 | 2236 |
| IRT | 1993967 | 38.9 | 1956 |
| KF-10 | 1948024 | 38.66 | 1899 |
| KF-1 | 1948653 | 38.65 | 1904 |
| L11 | 1937923 | 38.91 | 1823 |
| L1604-1 | 2119233 | 38.27 | 1977 |
| L8 | 2050876 | 38.62 | 2036 |
| LL7 | 2384717 | 38.81 | 2244 |
| lpuph1 | 2114327 | 38.38 | 2008 |
| Lr4000 | 2391171 | 38.55 | 254 |
| LRA7 | 2020909 | 38.75 | 1978 |
| LTH2584 | 2066054 | 38.53 | 2082 |
| LTH5448 | 1980298 | 38.44 | 1898 |
| LTM-Sauna-1-1 | 2186597 | 38.65 | 2042 |
| M1 | 2281552 | 38.82 | 2195 |
| M2021619 | 2229645 | 39.07 | 2158 |
| MD207 | 2014210 | 38.56 | 1917 |
| MGG32-12 | 2161400 | 38.7 | 2143 |
| MGG32-1 | 2132496 | 38.59 | 2084 |
| MH-1-1 | 1941848 | 38.7 | 1916 |
| MQZ-14 | 1900928 | 38.62 | 1853 |
| MQZ-15 | 1908235 | 38.62 | 1866 |
| N2J | 2112985 | 38.46 | 2021 |
| N4I | 2085822 | 38.58 | 1949 |
| NL02 | 2111986 | 38.81 | 2046 |
| NM167-4 | 2025722 | 38.9 | 1966 |
| One-one | 2156208 | 38.52 | 2023 |
| P43 | 2151063 | 38.76 | 2005 |
| PB-W1 | 2158617 | 38.54 | 1956 |
| PM-Patrick-2-1 | 2188188 | 38.63 | 2036 |
| PNG008-24h | 2154690 | 38.53 | 1956 |
| PNG008-48h | 2159046 | 38.54 | 1955 |
| PNG008-ANA | 2230458 | 38.57 | 2012 |
| PNG008C-M | 2158974 | 38.54 | 1955 |
| R2lc | 2091003 | 38.46 | 1980 |
| RE225 | 2280129 | 38.65 | 2192 |
| RTR | 1986363 | 38.89 | 1989 |
| SC103-10 | 2006520 | 38.82 | 2026 |
| SC103-11 | 2029667 | 38.75 | 2027 |
| SC103-14 | 1957894 | 38.87 | 1922 |
| SC50-4 | 2059815 | 38.74 | 2078 |
| SC50-5 | 1958465 | 38.72 | 1985 |
| SC58-10 | 2055574 | 38.77 | 2085 |
| SC58-11 | 2025972 | 38.67 | 2075 |
| SC58-12 | 2077898 | 38.71 | 2089 |
| SC58-1 | 1963110 | 38.77 | 1982 |
| SC58-2 | 2030996 | 38.67 | 2071 |
| SC58-3 | 2102562 | 38.8 | 2116 |
| SC58-4 | 1964766 | 38.67 | 1995 |
| SC58-5 | 1995898 | 38.63 | 2034 |
| SC58-6 | 2102497 | 38.8 | 2115 |
| SC58-7 | 2020326 | 38.75 | 2054 |
| SC58-8 | 2038799 | 38.78 | 2066 |
| SC58-9 | 2102007 | 38.8 | 2117 |
| SC74-2 | 2051089 | 38.81 | 2072 |
| SC74-5 | 2146391 | 38.98 | 2063 |
| SC78-17 | 2095365 | 38.8 | 2114 |
| SC78-18 | 2009588 | 38.74 | 2026 |
| SC78-19 | 1999603 | 38.75 | 2009 |
| SC78-20 | 2078926 | 38.86 | 2076 |
| SC78-4 | 2031698 | 38.77 | 2043 |
| SC78-5 | 1985138 | 38.75 | 1991 |
| SC78-7 | 2029353 | 38.83 | 2033 |
| SC79-8 | 1951947 | 38.81 | 1952 |
| SC82-6 | 2021532 | 39.39 | 1964 |
| SC82-8 | 1996297 | 39.12 | 2021 |
| SC83-14 | 1980302 | 38.95 | 2019 |
| SC84-12 | 2028596 | 38.96 | 2039 |
| SC84-17 | 1960001 | 38.8 | 1969 |
| SC84-6 | 2050432 | 39.48 | 2073 |
| SD2112 | 2316838 | 39.04 | 2281 |
| SD-LRE2-IT | 2313802 | 38.8 | 2424 |
| SD-RD830-FR | 2076814 | 38.92 | 2088 |
| SESE34-1 | 2204458 | 38.51 | 2096 |
| SKKU-OGDONS-01 | 2259968 | 38.86 | 2086 |
| SRCM210547 | 2244109 | 39.07 | 2224 |
| SRCM217606 | 2151824 | 38.92 | 2093 |
| SRCM217607 | 2157807 | 38.95 | 2086 |
| SRCM217608 | 2167327 | 38.83 | 2117 |
| SRCM217611 | 2093158 | 39 | 2023 |
| SRCM217616 | 2159049 | 38.95 | 2093 |
| SRCM217617 | 2206089 | 38.95 | 2136 |
| TK4-3 | 1955683 | 38.7 | 1979 |
| TK-F8A | 2038911 | 38.88 | 2014 |
| TMW1.112 | 2032034 | 38.45 | 1999 |
| TMW1.656 | 1949539 | 38.49 | 1993 |
| TPC32 | 2214495 | 38.81 | 2212 |
| UMB5704 | 2166252 | 38.82 | 1991 |
| VA24-5 | 2177265 | 38.77 | 2083 |
| VHProbiE18 | 2040678 | 38.88 | 2015 |
| VHProbiM07 | 2041530 | 38.89 | 2012 |
| VHProbiV43 | 2053710 | 38.88 | 2033 |
| WF-AA1-A | 2014409 | 38.38 | 1859 |
| WF-AA1-C | 2013591 | 38.38 | 1859 |
| WHH1689 | 2044184 | 39.31 | 2081 |
| WL62-20 | 2010487 | 38.78 | 2000 |
| XGMJ21-2-1 | 2150421 | 38.52 | 2046 |
| XGMJ21-2 | 2164183 | 38.57 | 2046 |
| XMB29-10 | 2010420 | 38.8 | 2017 |
| XMB29-14 | 1969543 | 38.76 | 1997 |
| XMB29-17 | 2068004 | 38.83 | 2057 |
| XMB29-18 | 1951303 | 38.75 | 1967 |
| XMB29-19 | 1960867 | 38.76 | 1971 |
| XMB29-1 | 1927562 | 38.73 | 1921 |
| XMB29-2 | 1969687 | 38.73 | 1958 |
| XMB29-3 | 1959977 | 38.77 | 1967 |
| XMB29-4 | 1965464 | 38.75 | 1991 |
| XMB29-6 | 1949538 | 38.68 | 1959 |
| XMB29-7 | 1958390 | 38.78 | 1969 |
| XMB29-9 | 1970972 | 38.75 | 2000 |
| XMB56-8 | 2131252 | 38.73 | 2033 |
| XMB56-9 | 2089797 | 38.66 | 2028 |
| YC10-11 | 1981080 | 38.73 | 1984 |
| YC10-12 | 1991834 | 38.74 | 1983 |
| YC10-13 | 1915843 | 38.8 | 1902 |
| YC10-19 | 1862771 | 38.79 | 1872 |
| YC10-3 | 1992587 | 38.74 | 1987 |
| YC10-4 | 1992998 | 38.74 | 1991 |
| YC10-5 | 1974769 | 38.75 | 1950 |
| YC10-6 | 1899913 | 38.73 | 1921 |
| YC10-7 | 1863144 | 38.79 | 1864 |
| YC10-8 | 1952215 | 38.71 | 1971 |
| YC10-9 | 1939354 | 38.72 | 1958 |
| YLR001 | 2439358 | 38.66 | 2447 |
| YSJL-12 | 2151788 | 38.93 | 2117 |
| ZLR003 | 2234097 | 38.66 | 2176 |

**Table S3. Drug resistance genes, homology, and sources of *Limosilactobacillus reuteri* genomes**

| **Genome ID** | **Drug resistance gene** | **Homology** | **source** |
| --- | --- | --- | --- |
| 03 | *AAC6_Ie_APH2_Ia* | 100 | Human intestinal tract |
| 121 | *ErmB* | 98.776 | Mammal |
| 121 | *ErmB* | 98.776 | Mammal |
| 121 | *vatE* | 100 | Mammal |
| 121 | *vatE* | 92.727 | Mammal |
| 121 | *tet(W)* | 96.87 | Mammal |
| 121 | *tet(O/W)* | 95.462 | Mammal |
| 121 | *tet(W/N/W)* | 94.671 | Mammal |
| 121 | *tet(O/W/O)* | 92.476 | Mammal |
| 121 | *tet(W)* | 96.87 | Mammal |
| 121 | *tet(O/W)* | 95.462 | Mammal |
| 121 | *tet(W/N/W)* | 94.671 | Mammal |
| 121 | *tet(O/W/O)* | 92.476 | Mammal |
| 121 | *tet(O/W)* | 93.165 | Mammal |
| 121 | *tet(O/W/O)* | 93.165 | Mammal |
| 121 | *tet(W)* | 95.167 | Mammal |
| 121 | *tet(O/W/32/O)* | 90.288 | Mammal |
| 121 | *tet(W/N/W)* | 91.078 | Mammal |
| 121 | *tet(O/W)* | 96.218 | Mammal |
| 121 | *tet(W/N/W)* | 97.046 | Mammal |
| 121 | *tet(W)* | 95.378 | Mammal |
| 121 | *tet(W/32/O)* | 92.017 | Mammal |
| 3630 | *tet(W)* | 96.714 | Birds |
| 3630 | *tet(O/W)* | 95.305 | Birds |
| 3630 | *tet(W/N/W)* | 94.357 | Birds |
| 3630 | *tet(O/W/O)* | 92.32 | Birds |
| 3632 | *tet(W)* | 96.714 | Birds |
| 3632 | *tet(O/W)* | 95.305 | Birds |
| 3632 | *tet(W/N/W)* | 94.357 | Birds |
| 3632 | *tet(O/W/O)* | 92.32 | Birds |
| 3c6 | *ErmB* | 98.776 | Mammal |
| 3c6 | *tet(W)* | 97.183 | Mammal |
| 3c6 | *tet(O/W)* | 95.618 | Mammal |
| 3c6 | *tet(W/N/W)* | 94.828 | Mammal |
| 3c6 | *tet(O/W/O)* | 92.476 | Mammal |
| AN417 | *fexA* | 97.474 | Birds |
| AN417 | *tet(M)* | 98.131 | Birds |
| CNEI-KCA3 | *Lreu_cat-TC* | 99.024 | Birds |
| CNEI-KCA3 | *Ssui_ACT_CHL* | 94.907 | Birds |
| CNEI-KCA3 | *ErmB* | 98.387 | Birds |
| CNEI-KCA3 | *tet(W)* | 96.238 | Birds |
| CNEI-KCA3 | *tet(O/W)* | 94.828 | Birds |
| CNEI-KCA3 | *tet(W/N/W)* | 93.887 | Birds |
| CNEI-KCA3 | *tet(O/W/O)* | 92.163 | Birds |
| CNEI-KCA3 | *lnuA* | 97.516 | Birds |
| HC5-4 | *ErmB* | 97.959 | Fermented foods |
| I5007 | *tet(W)* | 96.465 | Mammal |
| I5007 | *tet(O/W)* | 95.791 | Mammal |
| I5007 | *tet(W/N/W)* | 93.929 | Mammal |
| I5007 | *tet(O/W/O)* | 92.749 | Mammal |
| I5007 | *Ssui_ACT_CHL* | 96.296 | Mammal |
| I5007 | *Lreu_cat-TC* | 97.561 | Mammal |
| L8 | *tet(W)* | 97.183 | Ruminants |
| L8 | *tet(O/W)* | 95.618 | Ruminants |
| L8 | *tet(W/N/W)* | 94.828 | Ruminants |
| L8 | *tet(O/W/O)* | 92.476 | Ruminants |
| Lr4000 | *tet(W)* | 97.027 | Rodents |
| Lr4000 | *tet(O/W)* | 95.618 | Rodents |
| Lr4000 | *tet(W/N/W)* | 94.671 | Rodents |
| Lr4000 | *tet(O/W/O)* | 92.476 | Rodents |
| LRA7 | *lnuC* | 98.171 | Mammal |
| LRA7 | *lnuC* | 98.171 | Mammal |
| NL02 | *lnuA* | 97.516 | Human intestinal tract |
| SD2112 | *tet(W)* | 96.87 | Human intestinal tract |
| SD2112 | *tet(O/W)* | 95.462 | Human intestinal tract |
| SD2112 | *tet(W/N/W)* | 94.514 | Human intestinal tract |
| SD2112 | *tet(O/W/O)* | 92.476 | Human intestinal tract |
| SD2112 | *lnuA* | 97.516 | Human intestinal tract |
| SD-LRE2-IT | *vatE* | 100 | Human intestinal tract |
| SRCM210547 | *tet(W)* | 97.027 | Ruminants |
| SRCM210547 | *tet(O/W)* | 95.618 | Ruminants |
| SRCM210547 | *tet(W/N/W)* | 94.671 | Ruminants |
| SRCM210547 | *tet(O/W/O)* | 92.476 | Ruminants |
| SRCM217606 | *tet(W)* | 96.87 | Ruminants |
| SRCM217606 | *tet(O/W)* | 95.462 | Ruminants |
| SRCM217606 | *tet(W/N/W)* | 94.828 | Ruminants |
| SRCM217606 | *tet(O/W/O)* | 92.32 | Ruminants |
| SRCM217607 | *tet(W)* | 96.87 | Ruminants |
| SRCM217607 | *tet(O/W)* | 95.462 | Ruminants |
| SRCM217607 | *tet(W/N/W)* | 94.828 | Ruminants |
| SRCM217607 | *tet(O/W/O)* | 92.32 | Ruminants |
| SRCM217616 | *tet(W)* | 96.87 | Ruminants |
| SRCM217616 | *tet(O/W)* | 95.462 | Ruminants |
| SRCM217616 | *tet(W/N/W)* | 94.828 | Ruminants |
| SRCM217616 | *tet(O/W/O)* | 92.32 | Ruminants |
| TPC32 | *vatE* | 100 | Mammal |
| TPC32 | *tet(W)* | 92.476 | Mammal |
| TPC32 | *tet(O/W/O)* | 91.693 | Mammal |
| TPC32 | *tet(W/N/W)* | 90.439 | Mammal |
| TPC32 | *tet(O/W)* | 90.596 | Mammal |
| XMB29-10 | *lnuA* | 99.379 | Dairy products |
| XMB29-17 | *lnuA* | 99.379 | Dairy products |
| XMB56-8 | *ErmB* | 98.776 | Dairy products |
| XMB56-9 | *ErmB* | 98.776 | Dairy products |
| YSJL-12 | *tet(W)* | 97.022 | Human intestinal tract |
| YSJL-12 | *tet(O/W)* | 95.611 | Human intestinal tract |
| YSJL-12 | *tet(W/N/W)* | 94.514 | Human intestinal tract |
| YSJL-12 | *tet(O/W/O)* | 92.476 | Human intestinal tract |
| YSJL-12 | *vatE* | 97.196 | Human intestinal tract |
| ZLR003 | *tet(W)* | 96.87 | Mammal |
| ZLR003 | *tet(O/W)* | 95.462 | Mammal |
| ZLR003 | *tet(W/N/W)* | 94.828 | Mammal |
| ZLR003 | *tet(O/W/O)* | 92.32 | Mammal |
| ZLR003 | *tet(L)* | 98.472 | Mammal |
| ZLR003 | *dfrG* | 100 | Mammal |

**Table S4. Bacteriocin regions, classes, and sources of *Limosilactobacillus reuteri* genomes**

| Genome ID | Genomic Start (bp) | Genomic End (bp) | Class | Source |
| --- | --- | --- | --- | --- |
| 1B | 907325 | 927814 | 63.3;Enterolysin_A | Other |
|  | 1549997 | 1570429 | 64.3;Enterolysin_A | Other |
| 03 | 1631600 | 1652074 | 64.3;Enterolysin_A | Human intestinal tract |
| 2A | 35477 | 55954 | 64.3;Enterolysin_A | Mammal |
| 3c6 | 4189 | 15811 | Sactipeptide | Mammal |
| 19-E-3 | 597401 | 617833 | 64.3;Enterolysin_A | Mammal |
| 19-E-6 | 597422 | 617854 | 64.3;Enterolysin_A | Mammal |
| 100-23 | 510581 | 531022 | 64.3;Enterolysin_A | Rodents |
| 121 | 1496165 | 1516639 | 64.3;Enterolysin_A | Mammal |
|  | 6218 | 26218 | Sactipeptides | Mammal |
| 3630 | 2213323 | 2233770 | 64.3;Enterolysin_A | Birds |
| 3632 | 121181 | 141628 | 64.3;Enterolysin_A | Birds |
|  | 67457 | 88789 | 159.1;carnolysins | Birds |
|  | 1885919 | 1906414 | 63.3;Enterolysin_A | Birds |
|  | 58839 | 79271 | 64.3;Enterolysin_A | Birds |
| AM-LB1 | 1418019 | 1438484 | 63.3;Enterolysin_A | Birds |
| AN417 | 874049 | 894595 | 64.3;Enterolysin_A | Birds |
| ATCCPTA4659 | 1976561 | 1997026 | 62.3;enterolysin_A | Other |
| ATG-F4 | 1 | 14275 | 63.3;Enterolysin_A | Human intestinal tract |
| BHM-Lincecum-1-1 | 1 | 19600 | 7.2;Acidocin_LF221B(GassericinK7B) | Mammal |
| BHM-Lincecum-2-1 | 70211 | 90679 | 63.3;Enterolysin_A | Mammal |
| BHM-Thaddeus-3-1 | 96380 | 116818 | 63.3;Enterolysin_A | Mammal |
| BR13-C2 | 0 | 17111 | 64.3;Enterolysin_A | Rodents |
|  | 40428 | 60869 | 64.3;Enterolysin_A | #N/A |
| Byun-re-01 | 637160 | 1657607 | 64.3;Enterolysin_A | Rodents |
| CNI-KCA2 | 920477 | 940939 | 63.3;Enterolysin_A | Birds |
|  | 1421387 | 1441819 | 64.3;Enterolysin_A | Birds |
| C.p.1-C | 240842 | 261316 | 64.3;Enterolysin_A | Mammal |
| D.l.1-B | 240842 | 261316 | 64.3;Enterolysin_A | Rodents |
| D.l.3-A | 11788 | 32220 | 64.3;Enterolysin_A | Rodents |
| DS0384 | 166538 | 187012 | 64.3;Enterolysin_A | Human intestinal tract |
| DSM20016 | 7907 | 19420 | 62.3;enterolysin_A | Other |
| EFEL6901 | 483162 | 503627 | 62.3;enterolysin_A | Other |
| FN041 | 873707 | 894196 | 62.3;enterolysin_A | Human intestinal tract |
| I49 | 1058634 | 1079066 | 64.3;Enterolysin_A | Rodents |
| I5007 | 597344 | 617818 | 64.3;Enterolysin_A | Mammal |
| I8-5 | 5279 | 35720 | 63.3;Enterolysin_A | Rodents |
| IM842 | 110032 | 130473 | 64.3;Enterolysin_A | Human intestinal tract |
| IRT | 1496165 | 1516639 | 64.3;Enterolysin_A | Human intestinal tract |
|  | 6218 | 26218 | Sactipeptides | Human intestinal tract |
| L1604-1 | 200015 | 220474 | 64.3;Enterolysin_A | Rodents |
|  | 40407 | 60848 | 64.3;Enterolysin_A | Rodents |
| L8 | 1980110 | 2000656 | 64.3;Enterolysin_A | Ruminants |
| LL7 | 635693 | 656167 | 64.3;Enterolysin_A | Other |
| LRA7 | 1321273 | 1341729 | 63.3;Enterolysin_A | Mammal |
| LTM-Sauna-1-1 | 0 | 12110 | 62.3;enterolysin_A | Mammal |
| M1 | 810087 | 830519 | 64.3;Enterolysin_A | Rodents |
| M2021619 | 855272 | 875713 | 64.3;Enterolysin_A | Human intestinal tract |
| MD207 | 45302 | 57788 | 64.3;Enterolysin_A | Rodents |
|  | 119639 | 139639 | Sactipeptides | Rodents |
| N2J | -7231 | 12769 | Sactipeptides | Rodents |
|  | 0 | 18125 | 64.3;Enterolysin_A | Rodents |
|  | 52592 | 73066 | 64.3;Enterolysin_A | Rodents |
| N4I | -7921 | 19519 | Sactipeptides | Rodents |
|  | 43483 | 63972 | 63.3;Enterolysin_A | Rodents |
| NL02 | 1437231 | 1457672 | 64.3;Enterolysin_A | Human intestinal tract |
| P43 | 42711 | 63143 | 64.3;Enterolysin_A | Birds |
|  | 55707 | 76100 | 7.2;Acidocin_LF221B(GassericinK7B) | Birds |
| PB-W1 | 30952 | 51393 | 64.3;Enterolysin_A | Human intestinal tract |
|  | 85465 | 105954 | 62.3;enterolysin_A | Human intestinal tract |
| PM-Patrick-2-1 | 41858 | 53968 | 64.3;Enterolysin_A | Mammal |
| PNG008-24h | 16820 | 37309 | 63.3;Enterolysin_A | Human intestinal tract |
|  | 58388 | 78862 | 64.3;Enterolysin_A | Human intestinal tract |
| PNG008-48h | 85465 | 105954 | 63.3;Enterolysin_A | Human intestinal tract |
|  | 30952 | 51393 | 64.3;Enterolysin_A | Human intestinal tract |
| PNG008-ANA | 30951 | 51392 | 64.3;Enterolysin_A | Human intestinal tract |
|  | 16820 | 37309 | 62.3;enterolysin_A | Human intestinal tract |
| PNG008C-M | 16820 | 37309 | 62.3;enterolysin_A | Human intestinal tract |
|  | 30952 | 51393 | 64.3;Enterolysin_A | Human intestinal tract |
| R2lc | 26399 | 37757 | 64.3;Enterolysin_A | Rodents |
|  | 51806 | 71806 | Sactipeptides | Rodents |
| SD2112 | 384406 | 404847 | 64.3;Enterolysin_A | Human intestinal tract |
| SD-LRE2-IT | 1258323 | 1278788 | 63.3;Enterolysin_A | Human intestinal tract |
| SD-RD830-FR | 1575105 | 1595546 | 64.3;Enterolysin_A | Human intestinal tract |
| SKKU-OGDONS-01 | 1418019 | 1438484 | 63.3;Enterolysin_A | Birds |
| SRCM210547 | 1996882 | 2017428 | 64.3;Enterolysin_A | Ruminants |
| SRCM217606 | 855272 | 875713 | 64.3;Enterolysin_A | Ruminants |
| SRCM217607 | 1056499 | 1076988 | 62.3;enterolysin_A | Ruminants |
|  | 649538 | 669970 | 64.3;Enterolysin_A | Ruminants |
| SRCM217608 | 622547 | 642979 | 64.3;Enterolysin_A | Ruminants |
| SRCM217616 | 2115512 | 2136001 | 63.3;Enterolysin_A | Ruminants |
|  | 381061 | 401493 | 64.3;Enterolysin_A | Ruminants |
| SRCM217617 | 675086 | 695518 | 64.3;Enterolysin_A | Ruminants |
| TPC32 | 1 | 18022 | 63.3;Enterolysin_A | Mammal |
| UMB5704 | 286979 | 305758 | 62.3;enterolysin_A | Human intestinal tract |
| VHProbiE18 | 900392 | 920857 | 63.3;Enterolysin_A | Human intestinal tract |
| VHProbiM07 | 1201730 | 1222195 | 62.3;enterolysin_A | Ruminants |
| YSJL-12 | 574634 | 595108 | 64.3;Enterolysin_A | Human intestinal tract |
| ZLR003 | 2104979 | 2125438 | 64.3;Enterolysin_A | Mammal |
| BIO7251 | 0 | 10636 | 64.3;Enterolysin_A | Other |
| CRL1098 | 130604 | 151069 | 64.3;Enterolysin_A | Fermented foods |
|  | 0 | 19713 | 62.3;enterolysin_A | Fermented foods |
| HC5-4 | 25388 | 45862 | 64.3;Enterolysin_A | Fermented foods |
| IMAUJBR3 | 1476582 | 1497014 | 64.3;Enterolysin_A | Fermented foods |
| LTH2584 | 147062 | 167494 | 64.3;Enterolysin_A | Fermented foods |
|  | -7525 | 12475 | Sactipeptides | Fermented foods |
| TK-F8A | 1199096 | 1219561 | 63.3;Enterolysin_A | Dairy products |
| TMW1.656 | 199289 | 219289 | Sactipeptides | Fermented foods |
|  | 0 | 13869 | 64.3;Enterolysin_A | Fermented foods |
| VHProbiV43 | 914855 | 935320 | 62.3;enterolysin_A | Fermented foods |
